# The influence of energy and temperature distributions on EHD destabilization of an Oldroyd-B liquid jet

**DOI:** 10.1038/s41598-023-43157-z

**Published:** 2023-09-26

**Authors:** Galal M. Moatimid, Mohamed F. E. Amer

**Affiliations:** Department of Mathematics, Faculty of Education, Ain Shams University, Roxy, Cairo, Egypt

**Keywords:** Fluid dynamics, Applied mathematics

## Abstract

This work examines the impact of an unchanged longitudinal electric field and the ambient gas on the EHD instability of an Oldroyd-B fluid in a vertical cylinder, where the system is immersed in permeable media. In order to explore the possible subject uses in thermo-fluid systems, numerous experimental and theoretical types of research on the subject are conducted. The main factors influencing the dispersion and stability configurations are represented by the energy and concentration equations. The linear Boussinesq approximating framework is recommended for further convenience. A huge growth in numerous physical and technical implications is what motivated this study. Using the standard normal modes of examination, the characteristics of velocity fields, temperature, and concentration are analyzed. The conventional stability results in a non-dimensional convoluted transcendental dispersion connection between the non-dimensional growth rate and all other physical parameters. The Maranogoni phenomenon, in which temperature and concentration distributions affect surface tension, has been addressed. It is observed that the intense electric field, the Prandtl numeral, the Lewis numeral, and the Lewis numeral velocity ratio have a stabilizing influence. As opposed to the Weber numeral, the Ohnesorge numeral, and the density ratio have a destabilizing influence.

## Introduction

The viscoelastic Oldroyd-B model significant role in geothermal, engineering and industrial enhancement motivated us to carry out this in-depth investigation. The methodology of the nonlinear technique depends mainly on solving the linear equations of motion and applying the appropriate nonlinear BCs. Engineers have been interested in the instability and atomizing issues with fluid jets for a very long time. Investigations were made into the mechanisms causing a non-Newtonian dielectric liquid jet flowing through a dielectric gas with a ST gradient to experience temporal EHD axisymmetric instability^1^. Using the proper BCs, the dispersion connection between the growth rate and wave number for the eight constant Oldroyd model was determined. The constitutive model that governs the rheological behavior of viscoelastic liquids was essential for its mathematical representation. Additionally, these liquids were separated into three categories, namely differential, proportion, and integral types. The Oldroyd-B liquid is a subcategory of the rate liquid that specifies something together with relaxation and retardation time features. Other experiments in the area of two-dimensional flow preparation with various non-Newtonian liquid properties were reported^2^. In practice, the liquid might be of three-dimensional. Therefore, scientists have looked at three-dimensional flow for various liquid configurations for supplementary information. Some related works about different numerical algorithms of such models were given^3^. The nonlinear stability of a vertical cylindrical interface between two Oldroyd-B prototypes was studied^4^. The current investigation is developed in the cooperation with the viscoelastic Oldroyd-B flow because of its importance in numerous areas. Because of the significant of the Oldroyd-B model, therefore, in light of the great implication of the Oldroyd-B viscoelastic fluid in diverse practical applications, the current work is analyzed with respect to this topic.

Because of the significance of the MHT, Hsieh formulated a simplified version of the MHT^5^. To address the issues of interfacial stability with MHT, a simpler formulation was provided. However, it was shown that the MHT impacted significantly the classical stability criterion for KHI. When the vapor was warmer than the liquid and both phases were contained between two cylindrical surfaces that were concentric with the interface, as well as when there is an MHT across the contact, the KHI of the cylindrical interface between the vapor and liquid phases of a fluid was examined^6^. It was concluded that the MHT would increase with KHI. The KHI of a restrained Oldroyd-B fluid film with MHT was examined^7^. It was found that the MHT destabilized the stability configuration. It is worth remembering that the previous studies in MHT considered the simplified VPT as well as the simplified formulation of Hsieh. Additionally, the MP effect on the stability profile was examined^8^. Throughout the current work, regardless of utilizing the VPT, we proceed as in our previous work together with employing the Navier–Stokes equations^8^. With MHT over the interface, an investigation of the stability of a vertical cylindrical perturbed surface was reported^9^. Both analytically and numerically, the nonlinear analysis stability criterion was met and analyzed. The effects of various physical elements were shown through a series of diagrams. It should be noted that many industrial applications, as well as scientific and technical instruments, depend on MHT. Therefore, the current work is examined the presence of this phenomenon.

In homogenous isotropic porous media, a stability analysis of liquid interfaces moving at uniform velocities was provided^10^. It was discovered that the liquid layer has no bearing on the crucial conditions separating stable disturbances from unstable ones. An annular viscous liquid jet travelling in an inviscid gas medium was subjected to a linear analysis for its temporal instability, involving the three limitation instances of a round liquid jet, a gas jet, and a plane liquid sheet^11^. It was demonstrated that the annular jet instability is always made worse by an ambient gas medium. Numerous widespread studies, see Fu et al.^12^ require scrutinizing the instability of fluid sheets and cylinders from a different point of view, however, without MHT. In light of their numerous uses in the chemical and industrial fields, thermal enhancement research was expected to increase in the modern era^13^. The hydrodynamic instability of Hartmann flow in the porous medium was taken into consideration. The findings were applied to photodynamic treatment, medication delivery systems, and anticancer delivery. It was investigated if a cylinder-shaped interface, which separates two homogeneous, incompressible, porous viscous flowing liquids, is linearly or nonlinearly stable^14^. Due to the numerous applications of immiscible liquids, the MHT outcome in this study was challenging. The current study explores the interaction between an interfacial nonlinear stability with permeability and a constant tangential EF.

Both oil industry and hydrology depend on the instability of fluid interfaces flowing in permeable media. When the velocity of extraction was great, long tongues or cones of water penetrated the oil and came out with water in some types of oil fields. Pascal^15^ examined the rheological impact of streaming fluids of the non-Newtonian behavior on the instability of an interface in a permeable medium dividing two compressible and immiscible liquids. For more than a century, researchers have theoretically and experimentally examined the existence of a fluid flowing in porous media in a variety of situations. The nonlinear EHD instability of capillary gravitational oscillations, where the separation surface divides two semi-infinite dielectric permeable liquids were addressed^16^. Darcy’s coefficients were discovered to investigate the instability influence on the linear methodology. However, throughout the nonlinear approach, these coefficients together with the EF exert an influence on the instability configuration. The mixing of surface water and groundwater, as demonstrated^17^, was the most significant of these studies. In geology, technology, and biomechanics, the plane surface between viscous liquids through permeable media could be worthwhile. However, the linear stability of fluid-porous models has been well investigated, and the evaluation of the nonlinear stability methodology has a significant recent advancement. Owing to of the huge significance of the porous relationship, the existing study will be undertaken using this approach.

In view of the aforementioned aspects, the current study is performed. Therefore, the instability examination of a restricted viscoelastic annular fluid layer through a gas is considered. Away from the VPT, the full Navier–Stokes equation is analyzed. Additionally, the energy and concentration equations are supplemented to make up for the shortcoming^18^. Therefore, regardless of Hsieh’s simplification^5^, many parameters concerning the MHT are achieved. The current findings suggest that the MHT shows a significant role in the stability structure. Furthermore, an unchanging tangential EF is pervaded. A significant transcendental relationship is established in view of the typical regular examination. Non-dimensional physical numbers, as is well known, can investigate the backdrop of the movement of fluids. They also reduce the number of parameters needed to describe the procedure. These numbers are frequently having physical connotations that help explain various scientific phenomena. Therefore, non-dimensional approach yields several non-dimensional physical numerals. This examination is different from the previous studies; for example, Amer and Moatimid^18^ studied the viscous fluid jet in an inviscid gas medium with MHT using Hsieh^5^ simplification which reduces MHT in one parameter that appears only from the BCs and ignores the energy and concentration equations. Similarly, Moatimid et al.^19^ studied the fluid jet stability in the existence of heat transfer by only using the heat equation that depends on the MP effect and BA and using the VPF theory which considers the viscous flow only at the interface i.e., Euler equation of motion is used to obtain the solutions. The main goal of the current study is to guide readers in finding the right answers to the following questions:

- What is the benchmark of the stability methodology?
- How numerous physical non-dimensional numerals are present throughout the stability approach?
- What are the impacts of the non-dimensional physical numerals on the stability profile?

To make the article structure clearer, it will be constructed as: In Section “Theoretical outlines”, the organization of the manuscript is described. This Section introduces the principal equations in addition to the suitable BCs. The technique of explanation is also introduced. In Section “Dispersion connection”, the dispersion relation is introduced. The results and discussions are given in Section “Discussions and outcomes”. In Section “Concluding remarks”, the key outcomes of the work is summarized.

## Theoretical outlines

**Figure 1:**
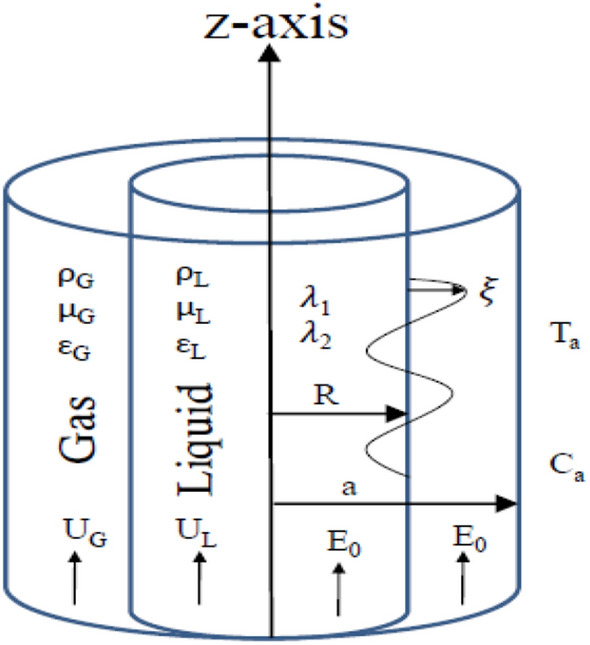
Sketch of the hypothetical prototypical.

The theoretical prototypical involves of two endless cylindrical movements. For the sake of simplicity and for more convenience, cylindrical polar coordinates are employed. The inner liquid inhabits a viscoelastic liquid, which guarantees the Oldroyd-B structure. The outer fluid cylinder is filled with perfect gas. One rigid cylinder is considered along with the outer fluid and is maintained at uniform temperature and concentration. Furthermore, a uniform axial EF effects the organization. Just for simplification, no surface currents are considered at the separating surface. The fluids are assumed to be immersed in permeable media with unit porosity. The gravitational force that works orthogonally downwards is considered. The ST is also considered, and the MP influence takes place. Figure 1 shows the shape of the two-phase flow layers which is modeled and discussed.

### Problem organization

The stability methodology inspiration is formed using the consistent style agreeing the pioneer work of Chandrasekhar^20^. Accordingly, any concerned function might be distributed as:

1 $$f(r,\,\theta ,\,z;t) = f(r)\exp \,\left( {i(kz + m\theta ) + \omega t} \right) + c.c.\,$$

where $f$ signifies any physical distribution.

The connection between the axial wave numeral $k$ and the disturbance of the wavelength $\lambda$ is $k = 2\pi /\lambda$. Additionally, the surface wave complex frequency is $\omega = \omega_{r} + i\omega_{i}$, where the real part $\omega_{r}$ is $2\pi$ times the disruption existence and $- \omega_{i} /k$ is the velocity spread in the fluid flow way. The wave numeral is considered as a positive real value in the examination of the temporal disturbance. Through the revision of temporal-spatial stability and the shift beyond convective absolute instability, both $k$ and $\omega$ are expected to be complex as: $\omega = \omega_{r} + i\omega_{i}$ and $k = k_{r} + ik_{i}$. Subsequently, $k_{i} > 0$ produces the spatial growth of instabilities supplementary with the stream direction**,** and $\omega_{r} > 0$ produces the time growth of instability^21,22^. Hypothetically, they displayed the temporal-spatial stability performance of an electrified viscoelastic fluid cylinder.

### Hydrodynamic of liquid part

The governing equations of liquid motion may be recorded.

The continuity equation consequences in^18^

2 $$\nabla .\,{\tilde{\mathbf{V}}}_{L} = 0$$

where ${\tilde{\mathbf{V}}}_{L}$ represents the liquid velocity vector.

The fundamental conservation of momentum of the fluid as^1^:

3 $$\rho_{L} \left( {\frac{{\partial {\tilde{\mathbf{V}}}_{L} }}{\partial t} + ({\tilde{\mathbf{V}}}_{L} .\nabla ){\tilde{\mathbf{V}}}_{L} } \right) = \nabla .\,{\tilde{\mathbf{\Pi }}}_{L} - \rho_{L} g\underline{{\mathbf{e}}}_{z} - \frac{{\eta_{0} }}{\kappa }{\tilde{\mathbf{V}}}_{L}$$

herein ${\tilde{\mathbf{\Pi }}}_{L}$ is the total stress tensor that was already given^1,23^ as:

4 $${\tilde{\mathbf{\Pi }}}_{L} = - \tilde{P}_{L} {{\varvec{\updelta}}}_{ij} + {\tilde{\mathbf{\tau }}}_{ij}$$

herein ${{\varvec{\updelta}}}_{ij}$ is referred to as the Kronecker delta and ${\tilde{\mathbf{\tau }}}_{{{\mathbf{ij}}}}$ represents the extra stress tensor.

As previously established^24,25^, the viscoelastic liquid state is designated by the Oldroyd 8-constant. After ignoring the non-linear parts and the gravitational effects, the following linearized equation is given:

5 $${\tilde{\mathbf{\tau }}} + \lambda_{1} \left( {\frac{\partial }{\partial t} + {\text{U}}_{L} \frac{\partial }{\partial z}} \right)\,{\tilde{\mathbf{\tau }}} = - \eta_{L} \left( {{\dot{\mathbf{\gamma }}} + \lambda_{2} \left( {\frac{\partial }{\partial t} + {\text{U}}_{L} \frac{\partial }{\partial z}} \right){\dot{\mathbf{\gamma }}}} \right)$$

The combination of Eq. (5) and the standard modes formula as agreed in Eq. (1) yields

6 $${\tilde{\mathbf{\tau }}} = - \eta (\omega ){\dot{\mathbf{\gamma }}}$$

where $$\eta (\omega ) = \eta_{L} \left( {\frac{{1 + \lambda_{2} \left( {\omega + ik{\text{U}}_{L} } \right)}}{{1 + \lambda_{1} \left( {\omega + ik{\text{U}}_{L} } \right)}}} \right)$$.

On the other hand, the present examination throughout porous media is presumed to be homogeneous, filled with the surrounding liquid, and the Newtonian behavior follows the linear BA. Subsequently, the density fluctuation in the presence of temperature and concentration is included in the formula:$\rho_{L} = \rho_{0L} \left[ {1 - \beta_{TL} \left( {\tilde{T}_{L} - T_{0} } \right) - \beta_{CL} \left( {\tilde{C}_{L} - C_{0} } \right)} \right]$, where temperature and concentration are independent of both position and time. As a consequence, one can assume a simple situation in which density varies as a distribution of pressure, heat, and concentration. The term equation of “state” denotes to the connection between these amounts. The pressure effects on density may be disregarded. It is merely linearly needy on heat and concentration^25^. The BA is applied, which requires that a minor modification remain terminates unless it is multiplied by gravity acceleration, as stated in the conceptual model. As a conclusion, the aforementioned equations of state, the parameters $\beta_{TL}$ and $\beta_{CL}$ are small amounts that are set to zero unless the gravitational acceleration is elevated^26^. In view of the inclusion of MHT as well as the BA, the governing equations of motion include a combination of these distributions. Therefore, we are forced to analyze the equations of energy and concentration. As previously shown^27^, we obtain

7 $$(\rho c_{p} )_{fL} \left( {\frac{\partial }{\partial t} + {\tilde{\mathbf{V}}}_{L} .\nabla } \right)\tilde{T}_{L} = k_{fL} \nabla^{2} \tilde{T}_{L}^{{}}$$

8 $${\text{and}}\;\left( {\frac{\partial }{\partial t} + {\tilde{\mathbf{V}}}_{L} .\nabla } \right)\tilde{C}_{L} = D_{BL} \nabla^{2} \tilde{C}_{L} + \frac{{D_{TL} }}{{T_{0} }}\nabla^{2} \tilde{T}_{L}$$

here $(\rho c_{p} )_{fL}$ indicates the fluid heat capability, $k_{fL}$ is the current conductivity, the original temperature is $T_{0}$, the thermophoresis factor is $D_{TL}$, and the Brownian diffusion parameter is $D_{BL}$.

The inclusion of the normal mode methodology in Eq. (7) yields

9 $$r^{2} \frac{{d^{2} T_{L} }}{{dr^{2} }} + r\frac{{dT_{L} }}{dr} - \left( {S_{L}^{2} r^{2} + m^{2} } \right)T_{L} = 0$$

where $$S_{L}^{2} = k^{2} + \frac{{(\rho c_{p} )_{L} }}{{k_{fL} }}\left( {\omega + ikU_{L} } \right)$$.

Equation (9) indicates the well-known Bessel differential equation. In order to attain a bounded solution at the beginning as $r \to 0$, one catches the following solution:

10 $$T_{L} (r) = A_{1} I_{m} (S_{L} r)$$

where $A_{1}$ represents an independent quantity which can be calculated based on the constraints. Its value will be omitted to avoid the length of the paper.

On the other side, the concentration equation as given in Eq. (8) then becomes

11 $$r^{2} \frac{{d^{2} C_{L} }}{{dr^{2} }} + r\frac{{dC_{L} }}{dr} - \left( {n_{L}^{2} r^{2} + m^{2} } \right)C_{L} = \frac{{D_{TL} }}{{T_{0} D_{BL} }}\left( {k^{2} - S_{L}^{2} } \right)r^{2} T_{L}$$

where $$n_{L}^{2} = k^{2} + \frac{{\omega + ikU_{L} }}{{D_{BL} }}$$.

As previously said, the finite solution of concentration throughout the liquid phase is given as follows:

12 $$C_{L} (r) = B_{1} (n_{L} r) + C_{0} \alpha_{1} I_{m} (S_{L} r)$$

where $B_{1}$ is an arbitrary constant to be calculated after the suitable BCs and $\alpha_{1} = \frac{{A_{1} D_{TL} }}{{T_{0} D_{BL} }}\left( {\frac{{k^{2} - S_{L}^{2} }}{{S_{L}^{2} - n_{L}^{2} }}} \right)$.

In light of the distributions of energy and concentration equations that are given in Eqs. (10) and (12), and with the aid of the constitutive Oldroyrd-B equations, BA, and the normal mode approach, the divergence of Eq. (3) produces

13 $$r^{2} \frac{{d^{2} P_{L} }}{{dr^{2} }} + r\frac{{dP_{L} }}{dr} - \left( {k^{2} r^{2} + m^{2} } \right)P_{L} = ikr^{2} \rho_{0L} g\left[ {\beta_{TL} A_{1} I_{m} (rS_{L} ) + \beta_{CL} \left( {B_{1} I_{m} (rn_{L} ) + \alpha_{1} C_{0} I_{m} (rS_{L} )} \right)} \right]$$

which is a non-homogeneous Bessel differential equation.

The precise integration of Eq. (13) is obtained by the MS. The overall solution of Eq. (13) can be formulated as:

14 $$P_{L} (r) = D_{1} I_{m} (kr) + ik\rho_{0L} g\left\{ {\left( {\frac{{\beta_{TL} A_{1} + \beta_{CL} C_{0} \alpha_{1} }}{{S_{L}^{2} - k^{2} }}} \right)I_{m} (S_{L} r) + \frac{{\beta_{CL} B_{1} }}{{n_{L}^{2} - k^{2} }}I_{m} (n_{L} r)} \right\}$$

herein the random constant will be evaluated from the convenient BCs.

Recurring back to the essential Eq. (3), the governing equations in the velocity components lengthways with the cylindrical coordinates might be recorded as:

15 $$r^{2} \frac{{d^{2} {\text{V}}_{rL} }}{{dr^{2} }} + r\frac{{d{\text{V}}_{rL} }}{dr} - \left( {q_{L}^{2} r^{2} + m^{2} + 1} \right){\text{V}}_{rL} = \frac{{r^{2} }}{\eta (\omega )}\frac{{dP_{L} }}{dr} + 2im{\text{V}}_{\theta L}$$

16 $$r^{2} \frac{{d^{2} {\text{V}}_{\theta L} }}{{dr^{2} }} + r\frac{{d{\text{V}}_{\theta L} }}{dr} - \left( {q_{L}^{2} r^{2} + m^{2} + 1} \right){\text{V}}_{\theta L} = \frac{imr}{{\eta (\omega )}}P_{L} - 2im{\text{V}}_{rL}$$

17 $$r^{2} \frac{{d^{2} {\text{V}}_{zL} }}{{dr^{2} }} + r\frac{{d{\text{V}}_{zL} }}{dr} - \left( {q_{L}^{2} r^{2} + m^{2} } \right){\text{V}}_{zL} = \frac{{ikr^{2} }}{\eta (\omega )}P_{L} - \frac{{\rho_{0L} gr^{2} }}{\eta (\omega )}\left( {\beta_{TL} T_{L} + \beta_{CL} C_{L} } \right)$$

where $$q_{L}^{2} = k^{2} + \frac{{\rho_{0L} \left( {\omega + ik{\text{U}}_{L} } \right) + \eta_{L} /\kappa }}{\eta (\omega )}$$.

Using the solutions for the energy and concentration equations, in the previous equations, one gets the solutions as follows:

18 $${\text{V}}_{rL} = \left( {\frac{{kD_{1} I^{\prime}_{m} (kr)}}{{\eta (\omega )\left( {k^{2} - q_{L}^{2} } \right)}} - \frac{{ikD_{2} }}{{q_{L} }}I_{m - 1} (q_{L} r) + \frac{{mD_{3} }}{{rq_{L} }}I_{m} (q_{L} r)} \right) + \frac{{i\rho_{0L} g}}{{k^{3} \eta (\omega )}}\left( {S_{L} I^{\prime}_{m} (S_{L} r)\alpha_{2} + n_{L} I^{\prime}_{m} (n_{L} r)\alpha_{3} } \right)$$

19 $${\text{V}}_{\theta L} = i\left( {\frac{{mD_{1} I_{m} (kr)}}{{r\eta (\omega )\left( {k^{2} - q_{L}^{2} } \right)}} - \frac{ik}{{q_{L} }}D_{2} I_{m - 1} (q_{L} r) + D_{3} I^{\prime}_{m} (q_{L} r)} \right) - \frac{{\rho_{0L} gm}}{{k^{3} r\eta (\omega )}}\left( {I_{m} (S_{L} r)\alpha_{2} + I_{m} (n_{L} r)\alpha_{3} } \right)$$

20 $${\text{V}}_{zL} (r) = \frac{{ikD_{1} I_{m} (kr)}}{{\eta (\omega )\left( {k^{2} - q_{L}^{2} } \right)}} + D_{2} I_{m} (q_{L} r) - \frac{{\rho_{0L} g}}{{k^{4} \eta (\omega )}}\left\{ {S_{L}^{2} I_{m} (S_{L} r)\alpha_{2} + n_{L}^{2} I_{m} (n_{L} r)\alpha_{3} } \right\}$$

where the constant $D_{i} \,,\,i = 1,2,3$ will be evaluated from the convenient BCs. To avoid the length of the paper, the structures of the arbitrary constants have been excluded. The dash indicates the differentiation in regard to the arguments, the considerations $\alpha_{2}$ and $\alpha_{3}$ are defined as $\alpha_{2} = \frac{{k^{4} \left( {\beta_{TL} A_{1} + \beta_{CL} C_{0} \alpha_{1} } \right)}}{{\left( {n_{L}^{2} - q_{L}^{2} } \right)\left( {n_{L}^{2} - k^{2} } \right)}}$ and $\alpha_{3} = \frac{{k^{4} \beta_{CL} B_{1} }}{{\left( {n_{L}^{2} - q_{L}^{2} } \right)\left( {n_{L}^{2} - k^{2} } \right)}}$.

In light of the previous analysis, remember that.

- The non-existence of the influence of the heat and concentration contributions and the distributions of the velocity components as well as pressure result in the same distributions as given in the previous work^28^.
- The preceding profiles in the fluid phase are legal in the area $r \le R$. The existence of the coordinate $r$ in the denominator of the formula ${\text{V}}_{\theta L}$ does not source any inconvenience. As recognized from the straightforward calculus, one finds $\mathop {Lim}\limits_{x \to 0} \frac{{I_{m} (x)}}{x}\,$ is finite $\forall \,m > 0$.
- The solutions of the liquid and gas phases like velocity and pressure as given in Eqs. (14), (18)–(20) and (23)–(26) cover the solution early obtained^28,29^ by putting the MHT parameters to zero or by disregarding the gravity acceleration and taking $a \to \infty$ . In addition, the parameter $q_{L}$ in our paper is compatible with the parameter $s$ in El-Sayed et al.^28^. Similarly, when $\lambda_{1} = \lambda_{2} = 0$ (i.e.$El = 0$), the jet non-Newtonian liquid is converted to that of Newtonian liquids (at this condition, $\eta_{L} = \mu$ where $\mu$ is the dynamic viscosity of Newtonian liquid).

The following subsection is devoted to introducing the findings throughout the gas phase.

### Hydrodynamic of gas phase

The profiles of velocities, pressure, heat, and concentration distributions may be presented as follows, by comparable influences as illustrated in the fluid phase:

The solution to the energy equation is given as:

21 $$T_{G} (r) = A_{2} I_{m} (S_{G} r) + A_{3} K_{m} (S_{G} r)$$

where $S_{G}^{2} = k^{2} + \frac{{(\rho c_{p} )_{G} }}{{k_{fG} }}\left( {\omega + ikU_{G} } \right)$, and $A_{2}$, $A_{3}$ are constants that might be evaluated using the applicable BCs.

Consequently, the concentration distribution can be formulated as:

22 $$C_{G} (r) = B_{2} I_{m} (n_{G} r) + B_{3} K_{m} (n_{G} r) + C_{0} \left( {\alpha_{4} I_{m} (S_{G} r) + \alpha_{5} K_{m} (S_{G} r)} \right)$$

where $B_{2}$ and $B_{3}$ are constants that can be designed by the applicable BCs.

Additionally, one gets.

$$n_{G}^{2} = k^{2} + \frac{{\omega + ikU_{G} }}{{D_{BG} }},\;\;\alpha_{4} = \frac{{D_{TG} }}{{C_{0} D_{BG} }}\left( {\frac{{A_{2} }}{{T_{0} }}} \right)\left( {\frac{{k^{2} - S_{G}^{2} }}{{S_{G}^{2} - n_{G}^{2} }}} \right)\;\;{\text{and}}\;\;\alpha_{5} = \frac{{D_{TG} }}{{C_{0} D_{BG} }}\left( {\frac{{A_{3} }}{{T_{0} }}} \right)\left( {\frac{{k^{2} - S_{G}^{2} }}{{S_{G}^{2} - n_{G}^{2} }}} \right)$$

The components of the velocity distributions and the pressure are specified by

23 $${\text{V}}_{rG} (r) = - \frac{1}{{\eta_{G} q_{G}^{2} }}\left\{ {k\left( {D_{4} I^{\prime}_{m} (kr) + D_{5} K^{\prime}_{m} (kr)} \right) + ig\rho_{0G} \left( {\alpha_{6} I^{\prime}_{m} (S_{G} r) + \alpha_{7} K^{\prime}_{m} (S_{G} r) + \alpha_{8} I^{\prime}_{m} (n_{G} r) + \alpha_{9} K^{\prime}_{m} (n_{G} r)} \right)} \right\}$$

24 $${\text{V}}_{\theta G} (r) = - \frac{im}{{r\eta_{G} q_{G}^{2} }}\left\{ {D_{4} I_{m} (kr) + D_{5} K_{m} (kr) + ig\rho_{0G} \left( {\frac{{\alpha_{6} }}{{S_{G} }}I_{m} (S_{G} r) + \frac{{\alpha_{7} }}{{S_{G} }}K_{m} (S_{G} r) + \frac{{\alpha_{8} }}{{n_{G} }}I_{m} (n_{G} r) + \frac{{\alpha_{9} }}{{n_{G} }}K_{m} (n_{G} r)} \right)} \right\}$$

25 $$\begin{aligned} {\text{V}}_{zG} (r) = & - \frac{ik}{{\eta_{G} q_{G}^{2} }}\left\{ {D_{4} I_{m} (kr) + D_{5} K_{m} (kr) + ig\rho_{0G} \left( {\frac{{\alpha_{6} }}{{S_{G} }}I_{m} (S_{G} r) + \frac{{\alpha_{7} }}{{S_{G} }}K_{m} (S_{G} r) + \frac{{\alpha_{8} }}{{n_{G} }}I_{m} (n_{G} r) + \frac{{\alpha_{9} }}{{n_{G} }}K_{m} (n_{G} r)} \right)} \right\} + \\ & \frac{{g\rho_{0G} }}{{\eta_{G} q_{G}^{2} }}\left( {\beta_{TG} T_{G} (r) + \beta_{CG} C_{G} (r)} \right) \\ \end{aligned}$$

and

26 $$P_{G} (r) = D_{4} I_{m} (kr) + D_{5} K_{m} (kr) + ig\rho_{0G} \left( {\frac{{\alpha_{6} }}{{S_{G} }}I_{m} (S_{G} r) + \frac{{\alpha_{7} }}{{S_{G} }}K_{m} (S_{G} r) + \frac{{\alpha_{8} }}{{n_{G} }}I_{m} (n_{G} r) + \frac{{\alpha_{9} }}{{n_{G} }}K_{m} (n_{G} r)} \right)$$

where.

$$q_{G}^{2} = \frac{1}{\kappa } + \frac{{\rho_{0G} \left( {\omega + ikU_{G} } \right)}}{{\eta_{G} }}$$, $$\alpha_{6} = \frac{{kS_{G} \left( {\beta_{TG} A_{2} + \beta_{CG} C_{0} \alpha_{2} } \right)}}{{S_{G}^{2} - k^{2} }}$$, $$\alpha_{7} = \frac{{kS_{G} \left( {\beta_{TG} A_{3} + \beta_{CG} C_{0} \alpha_{3} } \right)}}{{S_{G}^{2} - k^{2} }}$$, $$\alpha_{8} = \frac{{kn_{G} \beta_{CG} B_{2} }}{{S_{G}^{2} - k^{2} }}$$, and $$\alpha_{9} = \frac{{kn_{G} \beta_{CG} B_{3} }}{{S_{G}^{2} - k^{2} }}.$$

The subsequent item is depicted to presenting the contribution of the external axial uniform EF.

### Impact of EF

The documented Maxwell’s formulae must be involved due to the apparent EF intensity on this situation^23^. Melcher^23^ presented an innovator book comprising a comprehensive examination of the surface waves of EHD and MHD. Presently, only the effect of axial EF strength is reflected. Therefore, the MF impact can be ignored. The Maxwell expressions are shortened to grounded on:

27 $$\nabla .\,\,\varepsilon_{j} {\tilde{\mathbf{E}}}_{j} = 0,\;\;\;\;\;\;j = L\,\,\,{\text{and}}\,\,\,G$$

and

28 $$\nabla \, \times {\tilde{\mathbf{E}}}_{j} = 0$$

As exposed in the methodology of the considered problem, the interface currents are disregarded. As a significance, a scalar electric potential $\tilde{\psi }_{j} (r,\,\theta ,\,\,z;t)$ might be working to designate the EF. The greedy perturbed EF could be formulated as:

29 $${\tilde{\mathbf{E}}}_{j} = \left( { - \frac{{\partial \tilde{\psi }_{j} }}{\partial r},\, - \frac{1}{r}\frac{{\partial \tilde{\psi }_{j} }}{\partial \theta },\,{\text{E}}_{0} - \frac{{\partial \tilde{\psi }_{j} }}{\partial z}} \right)$$

The disturbed electric potential distribution $\tilde{\psi }_{j}$ verifies the following well-known Laplace’s equation:

30 $$\nabla^{2} \tilde{\psi }_{j} (r,\theta ,\,\,z;t) = 0$$

As designated during the consistent mode inspection, one obtains

31 $$\tilde{\psi }(r,\,\theta ,\,z;t) = \psi (r)\exp \,\left( {i(kz + m\theta ) + \omega t} \right) + c.c.\,$$

The electric potential must fulfill the following equation, as Eq. (31) is implanted on Eq. (30) as follows:

32 $$r^{2} \frac{{d^{2} \psi_{j} }}{{dr^{2} }} + r\frac{{d\psi_{j} }}{dr} - \left( {k^{2} r^{2} + m^{2} } \right)\psi_{j} = 0$$

which provides the modified Bessel differential equation.

Consequently, the electric potential in the gas phase, for a finite solution, is given as

33 $$\psi_{L} (r) = F_{1} I_{m} (kr)$$

where $F_{1}$ represents the constant quantity which can be evaluated based on the BCs..

On the other hand, in the gas phase, one finds

34 $$\psi_{G} (r) = F_{2} I_{m} (kr) + F_{3} K_{m} (kr)$$

where $F_{2}$ and $F_{3}$ are coefficients to be addressed from the BCs.

Lastly, the following item is depicted to presenting the appropriate BCs.

### Appropriate BCs

The BCs lengthways the instability methodology involve velocities, temperatures, concentrations, and electric potential distributions. These circumstances can be characterized in two classes as follows:

- **At the solid boundary** $r = a$, one gets
  35 $${\text{V}}_{rG} = 0,\;\;T_{G} = T_{a} ,\;\;C_{G} = C_{a} \;\;{\text{and}}\;\;\frac{{\partial \psi_{G} }}{\partial r} = 0$$
- **At the perturbed interface at** $\xi = \xi_{0} \exp \,\left( {i(kz + m\theta ) + \omega t} \right) + c.c.\,$.
  - **For the energy distribution,** one produces
    36 $$T_{L} = T_{G} ,\;\;and\;\;k_{fL} \frac{{\partial T_{L} }}{\partial r} = k_{fG} \frac{{\partial T_{G} }}{\partial r}$$
  - **For the concentration distribution**, one catches
    37 $$C_{L} = C_{G} ,\;\;and\;\;D_{BL} \frac{{\partial C_{L} }}{\partial r} = D_{BG} \frac{{\partial C_{G} }}{\partial r}$$
  - **Regarding the hydrodynamic part**, one obtains.

In light of the MP effect, the surface tension can now be stated as follows:

38 $$\sigma = \sigma_{0} \left[ {1 - \gamma_{T} \left( {\tilde{T} - T_{0} } \right) - \gamma_{C} \left( {\tilde{C} - C_{0} } \right)} \right]$$

The remaining interfacial condition deal**s** with the shear stresses and their relationship with the gradient and the profile of the surface tension as follows:

39 $$- \tau_{rz} = \frac{1}{r}\frac{\partial \sigma }{{\partial z}}$$

40 $$- \tau_{r\theta } = \frac{1}{r}\frac{\partial \sigma }{{\partial \theta }}$$

41 $${\text{and}}\;\;\;\tau_{\theta z} = 0$$

The previous conditions are previously referenced^8,19,27,30^.

The following BCs occur at the perturbed interface, where the mass conservation flux yields

42 $$\begin{gathered} \rho _{{0L}} \left. {\frac{{dF}}{{dt}}} \right|_{{at\,L}} = \rho _{{0G}} \left. {\frac{{dF}}{{dt}}} \right|_{{at\,G}} ,\,\,\,F = r - \xi \left( {\theta ,\,z;\,t} \right) \hfill \\ {\text{or}} \hfill \\ \rho _{{0L}} \left( {\frac{{\partial F}}{{\partial t}} + \underline{{\tilde{V}}} _{L} .\nabla F} \right) = \rho _{{0G}} \left( {\frac{{\partial F}}{{\partial t}} + \underline{{\tilde{V}}} _{G} .\nabla F} \right) \hfill \\ \end{gathered}$$

Equation (42) gives an alternative condition to the kinematic condition which happens in the absence of the concentration distribution. This condition has been adopted in the literature; for instance, see Hsieh^5^, Amer and Moatimid^18^, Moatimid et al.^8^, and many others.

- **Concerning the electric part,** as shown by Melcher^23^, at the interface, there is
  43 $$\underline{n} \times \left\| {\mathbf{E}} \right\| = \underline{0}$$
  where the jump across the surface of separation is represented by $\left\| * \right\|$.

Additionally, one gets

44 $$\underline{n} \cdot \left\| {\varepsilon \,\,{\mathbf{E}}} \right\| = 0$$

## Dispersion connection

As initially established^23,31^, the whole stress tensor might be expressed as:

45 $${{\varvec{\Pi}}}_{ij} = P{{\varvec{\updelta}}}_{ij} - \eta (\omega )\left( {\frac{{\partial v_{i} }}{{\partial x_{j} }} + \frac{{\partial v_{j} }}{{\partial x_{i} }}} \right) + \varepsilon {\mathbf{E}}_{i} {\mathbf{E}}_{j} - \frac{1}{2}\varepsilon {\mathbf{E}}^{2} \delta_{ij}$$

Discussing to the usual stress BCs, the change in the standard stress tensor components among the liquids at the surface of separation is discontinued by the amount of the ST. Consequently, the dispersion connection might be recognized. The normal stress tensor at the zero-order produces:

46 $$\Gamma_{L} - \Gamma_{G} = \frac{{\sigma_{0} }}{R}\left( {1 + \gamma_{T} T_{0} + \gamma_{C} C_{0} } \right) + \frac{1}{2}(\varepsilon_{L} {\text{E}}_{0L}^{2} - \varepsilon_{G} {\text{E}}_{0G}^{2} )$$

Throughout the linear state, one gets

47 $$P_{L} - P_{G} - 2\eta \left( \omega \right)\frac{{\partial V_{rL} }}{\partial r} - {\text{E}}_{0} \left( {\varepsilon_{L} \frac{{\partial \psi_{L} }}{\partial z} - \varepsilon_{G} \frac{{\partial \psi_{G} }}{\partial z}} \right) + \frac{{\sigma_{0} \xi_{0} }}{{R^{2} }}\left[ {\left( {1 + \gamma_{T} T_{0} + \gamma_{C} C_{0} } \right)\left( {1 - m^{2} - k^{2} R^{2} } \right) + \frac{R}{{\xi_{0} }}H(R)} \right]\,,\,\,\,r = R + \xi$$

Equations (10), (12), (14), (18), (26), (33), (34) and (47) produces

48 $$\begin{gathered} \,\frac{1}{\Delta }\left( {ik^{2} R^{2} H\left( R \right)\Delta_{11} + R^{2} Z^{2} \Delta_{12} } \right)I_{m} (kR) + iZ^{2} R^{2} \left( {\frac{{k^{2} \left( {Gr_{T} \Delta_{1T} + Gr_{C} \alpha_{1} \Delta_{T} } \right)}}{{\left( {S_{L}^{2} - k^{2} } \right)\Delta_{T} }}I_{m} (S_{L} R) + \frac{{k^{2} Gr_{C} }}{{\left( {n_{L}^{2} - k^{2} } \right)}}I_{m} (n_{L} R)} \right) \hfill \\ - \frac{1}{{\Delta_{G} }}\left[ {\left( { - iR^{2} Z^{2} \Delta_{14} + ik^{2} R^{2} H\left( R \right)H_{4} K^{\prime}_{m} (ka) - R^{2} q_{G}^{2} \eta ZK^{\prime}_{m} (ka)\Omega } \right)I_{m} (kR) + \left( { - iR^{2} Z^{2} \Delta_{24} - ik^{2} R^{2} H\left( R \right)H_{4} I^{\prime}_{m} (ka)} \right.} \right. \hfill \\ \left. {\left. { + R^{2} q_{G}^{2} \eta ZI^{\prime}_{m} (ka)(ka)\Omega } \right)K_{m} (kR) + iR^{2} Z^{2} \rho_{0} \left. {\left( {\frac{{k\alpha_{6} }}{{S_{G} }}I_{m} (S_{G} R) + \frac{{k\alpha_{7} }}{{S_{G} }}K_{m} (S_{G} R)} \right. + \frac{{k\alpha_{8} }}{{n_{G} }}I_{m} (n_{G} R) + \frac{{k\alpha_{9} }}{{n_{G} }}K_{m} (n_{G} R)} \right)} \right] \hfill \\ - \frac{2}{\Delta }\left[ {\frac{{k^{2} I^{\prime\prime}_{m} (kR)}}{{k^{2} - q_{L}^{2} }}} \right.\left( {ik^{2} R^{2} H\left( R \right)\Delta_{11} + R^{2} Z^{2} \Delta_{12} } \right) + iI^{\prime}_{m} (q_{L} R)\left( {ik^{2} R^{2} H\left( R \right)\Delta_{21} + R^{2} Z^{2} \Delta_{22} } \right) \hfill \\ \left. { + \frac{m}{kR}I^{\prime}_{m} (q_{L} R)\left( {ik^{2} R^{2} H\left( R \right)\Delta_{31} + R^{2} Z^{2} \Delta_{32} } \right) + iR^{2} Z^{2} \Delta \left( {\frac{{S_{L}^{2} }}{{k^{2} }}I^{\prime\prime}_{m} (S_{L} R)\alpha_{2} + \frac{{n_{L}^{2} }}{{k^{2} }}I^{\prime\prime}_{m} (n_{L} R)\alpha_{3} } \right)} \right] - \frac{{k^{2} R^{2} E_{0}^{2} \left( {\varepsilon - 1} \right)^{2} \Delta_{1E} }}{{\Delta_{E} }} \hfill \\ + k\left( {1 + \gamma_{T} + \gamma_{C} } \right)\left( {1 - m^{2} - k^{2} R^{2} } \right) + kRH\left( R \right) = 0 \hfill \\ \end{gathered}$$

where $$\Omega = \frac{1}{\rho }\left[ {\left( {\omega + ik\sqrt {We} } \right) - \rho \left( {\omega + ikU\sqrt {We} } \right)} \right]$$, $$\Delta_{1E} = I_{m} (kR)\left( {I_{m} (kR)K^{\prime}_{m} (ka) - I^{\prime}_{m} (ka)K_{m} (kR)} \right)$$.

To decrease the size of the article, the arbitrary constants have been crossed out from the Appendix in such a way to make the manuscript concise, therefore, $\Delta_{ij}$ will be excluded. Furthermore, the star (*) shows for the non-dimensional factor.

It is worthy to note that our study addresses the governing equations of motion in a dimensional form, which is well-known. For the sake of straightforwardness, a non-dimensional analysis is only performed in the concluding formula of the normal stress tensor at the surface of separation. For greater reliability, all the material factors are communicated in a non-dimensional style. Therefore, the comming procedure provides a non-dimensional clarification for the dispersion connection. Founded on the length, duration time and mass parameters, the following methodology may have a diversity of paths. Furthermore, numerous non-dimensional parameters are taken into account.

$$R^{*} = R/\xi_{0}$$, $$q_{j}^{*} = q_{j} \xi_{0}$$, $$S_{j}^{*} = S_{j} \xi_{0}$$, $$n_{j}^{*} = n_{j} \xi_{0}$$, $$k^{*} = k\xi_{0}$$, $$q = q_{G} /q_{L}$$, $$S = S_{G} /S_{L}$$, $$n = n_{G} /n_{L}$$, $$k_{f} = k_{fG} /k_{fL}$$, $$D_{B} = D_{BG} /D_{BT}$$, $$D_{T} = D_{TG} /D_{TL}$$, $$\beta_{T} = \beta_{TG} /\beta_{TL}$$, $$\beta_{C} = \beta_{CG} /\beta_{CL}$$, $$\rho_{0} = \rho_{0G} /\rho_{0L}$$, $$c_{p} = c_{pG} /c_{pL}$$, $$\eta = \eta_{G} /\eta_{L}$$, $${\text{U}} = {\text{U}}_{G} /{\text{U}}_{L}$$, $$\lambda = \lambda_{2} /\lambda_{1}$$, $$\varepsilon = \varepsilon_{G} /\varepsilon_{L}$$, $$\gamma_{T}^{*} = \gamma_{T} T_{0}$$, $$\gamma_{C}^{*} = \gamma_{C} T_{0}$$, $$a^{*} = a/\xi_{0}$$, $$T_{a}^{*} = T_{a} /T_{0}$$, and $$C_{a}^{*} = C_{a} /C_{0}.$$

To avoid repetition, these non-dimensional numbers are previously shown in the Nomenclature Section, and they are mathematically defined as follows: $We = \rho_{0L} U_{L}^{2} \xi_{0} /\sigma_{0}$, $Z = \eta_{L} /\sqrt {\rho_{0L} \sigma_{0} \xi_{0} }$, $Da = \kappa /\xi_{0}^{2}$, $Gr_{T} = \rho_{0L}^{2} g\beta_{TL} T_{0} \xi_{0}^{3} /\eta_{L}^{2}$, $Gr_{T} = \rho_{0L}^{2} g\beta_{CL} C_{0} \xi_{0}^{3} /\eta_{L}^{2}$,$\Pr = \eta_{L} c_{pL} /k_{fL}$, $Le = k_{fL} /\rho_{0L} c_{{p_{L} }} D_{BL}$, $El = \lambda_{1} \eta_{L} /\rho_{0L} \xi_{0}^{2}$, $N_{a} = D_{TL} /D_{BL} C_{0}$, $E_{0}^{*2} = \varepsilon_{L} E_{0}^{2} \xi_{0} /\sigma_{0}$, $\omega {}^{*} = \omega \sqrt {\rho_{0L} \xi_{0}^{3} /\sigma_{0} }$.

Together with $S_{L}^{*2} = k^{*2} + \frac{{\Pr \,\left( {\omega^{*} + ik^{*} \sqrt {We} } \right)}}{Z}$, $S_{G}^{*2} = k^{*2} + \frac{{\rho_{0} c_{p} \Pr \,\left( {\omega^{*} + ik^{*} U\sqrt {We} } \right)}}{{k_{f} Z}}$, $n_{L}^{*2} = k^{*2} + \frac{{Le\,\Pr \,\left( {\omega^{*} + ik^{*} \sqrt {We} } \right)}}{Z}$, $n_{G}^{*2} = k^{*2} + \frac{{Le\Pr \,\left( {\omega^{*} + ik^{*} U\sqrt {We} } \right)}}{{D_{B} Z}}$, $q_{L}^{*2} = k^{*2} + \left( {\frac{{\,\left( {\omega^{*} + ik^{*} \sqrt {We} } \right)}}{Z} + \frac{1}{Da}} \right)\left( {\frac{{Z + El\,\left( {\omega^{*} + ik^{*} \sqrt {We} } \right)}}{{Z + \lambda El\,\left( {\omega^{*} + ik^{*} \sqrt {We} } \right)}}} \right)$, $q_{G}^{*2} = \frac{{\,\rho_{0} \left( {\omega^{*} + ik^{*} \sqrt {We} } \right)}}{\eta Z} + \frac{1}{Da}$, and $\eta \left( \omega \right) = \eta_{L} \left( {\frac{{Z + \lambda El\,\left( {\omega^{*} + ik^{*} \sqrt {We} } \right)}}{{Z + El\,\left( {\omega^{*} + ik^{*} \sqrt {We} } \right)}}} \right)$, where $\omega^{*} = \omega_{r}^{*} + i\sqrt {We} \omega_{i}^{*}$, anywhere $\omega_{r}^{*} = \omega_{r} \sqrt {\rho_{0L} \xi_{0}^{3} /\sigma_{0} }$ is a dimensionless growth amount and $\omega_{i}^{*} = \left( {\xi_{0} /{\text{U}}_{L} } \right)\omega_{i}$ is a dimensionless disturbed frequency.

Overall, the importance of the time constant ratio $\lambda = \lambda_{2} /\lambda_{1}$ is reflected to be lying between 1/9 and 1; for instance**,** see Bird et al.^24^. When $\lambda_{1} = \lambda_{2} = 0$ (i.e.$El = 0$), the non-Newtonian liquid jet is converted to that of the Newtonian liquids (at this situation $\eta_{L} = \mu$, somewhere $\mu$ is the dynamic viscosity of Newtonian liquid). Equation (48) represents the principal equation and constitutes the base of the current study. Consequently, the next Section is dedicated to presenting a calculation design for the dispersion relation. The purpose is to display the impacts of the several material values on the instability configuration.

## Discussions and outcomes

**Figure 2:**
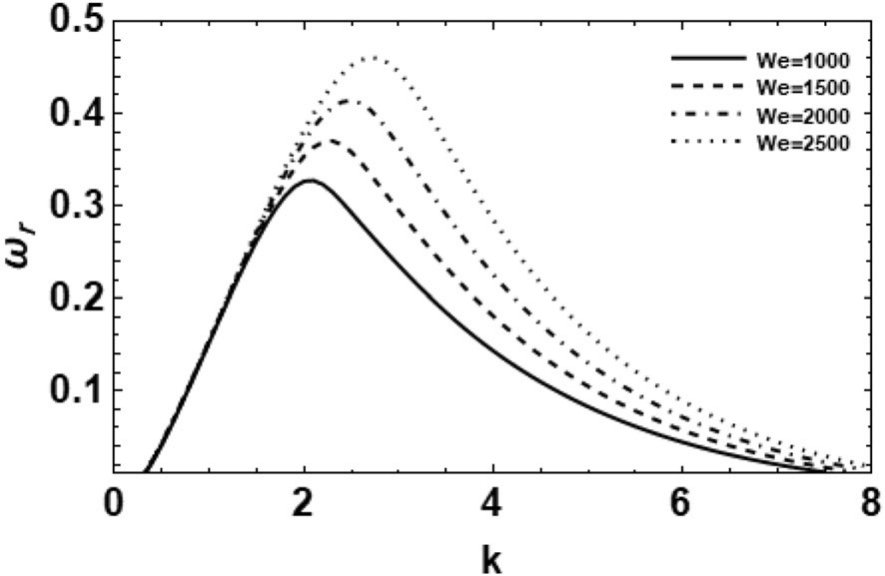
Depicts the real part of the surface wave frequency versus the wave number in light of Eq. (48) for different amounts of $We$.

**Figure 3:**
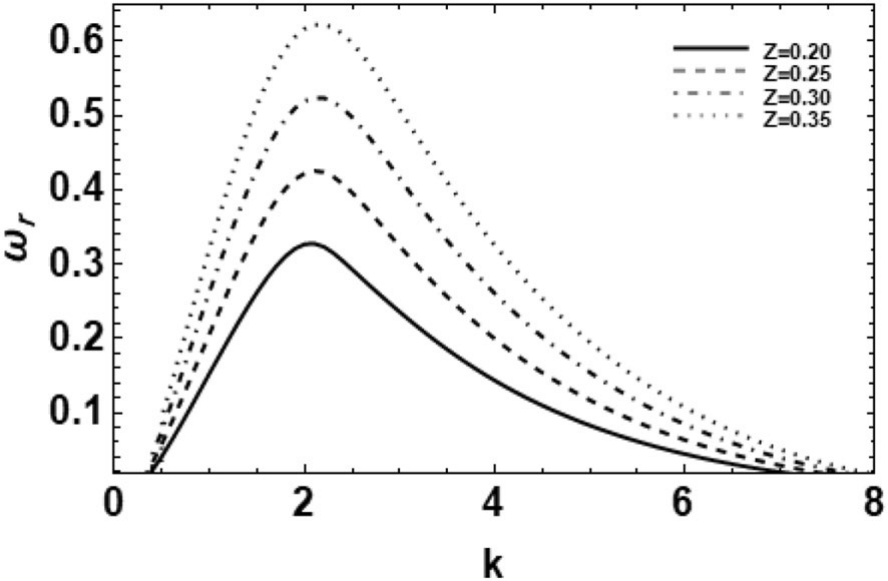
Depicts the real part of the surface wave frequency against the wave number in light of Eq. (48) for different amounts of $Z$.

**Figure 4:**
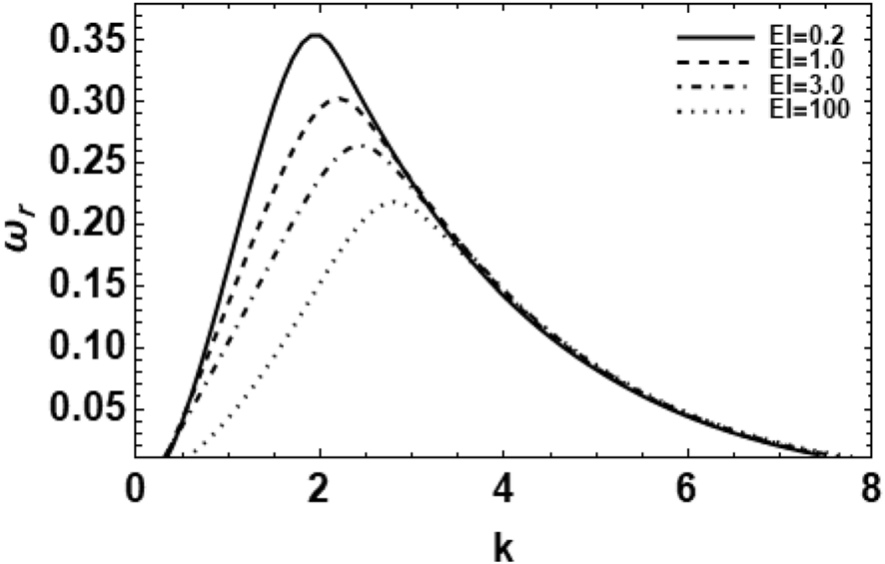
Shows the real part of the surface wave frequency versus the wave numeral in light of Eq. (48) for different amounts of $El$.

**Figure 5:**
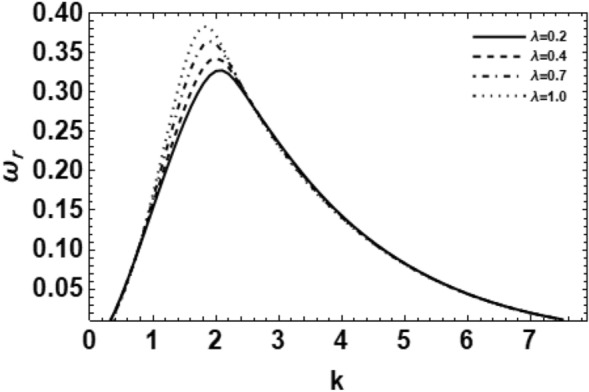
Shows the real part of the surface wave frequency against the wave number in light of Eq. (48) for different amounts of $\lambda$.

**Figure 6:**
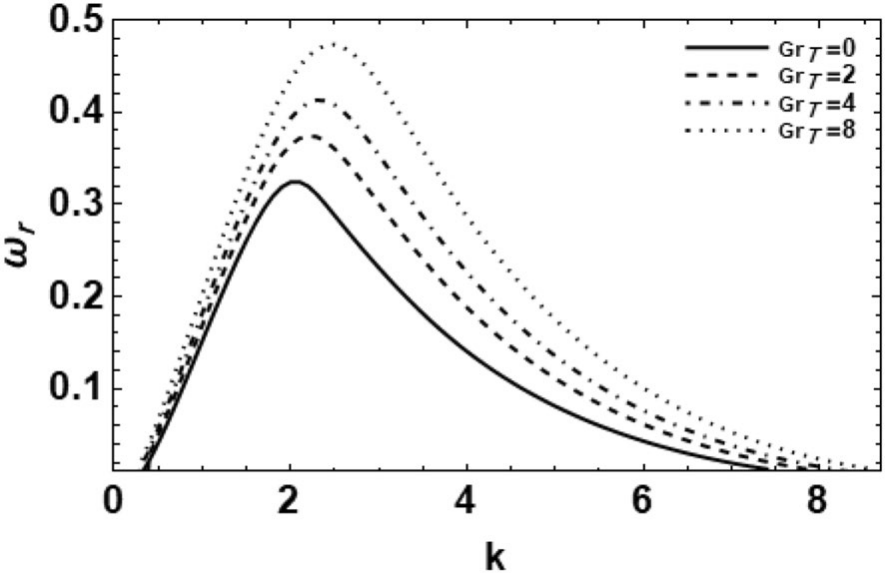
Shows the real part of the surface wave frequency against the wave numeral in light of Eq. (48) for different amounts of $Gr$.

**Figure 7:**
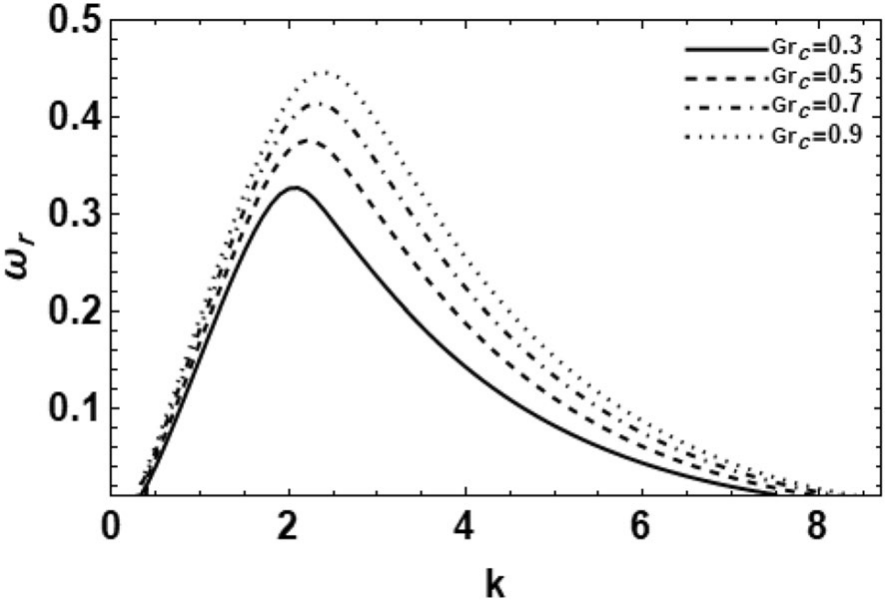
Shows the real part of the surface wave frequency against the wave numeral in light of Eq. (48) for different amounts of $Gr_{c}$.

**Figure 8:**
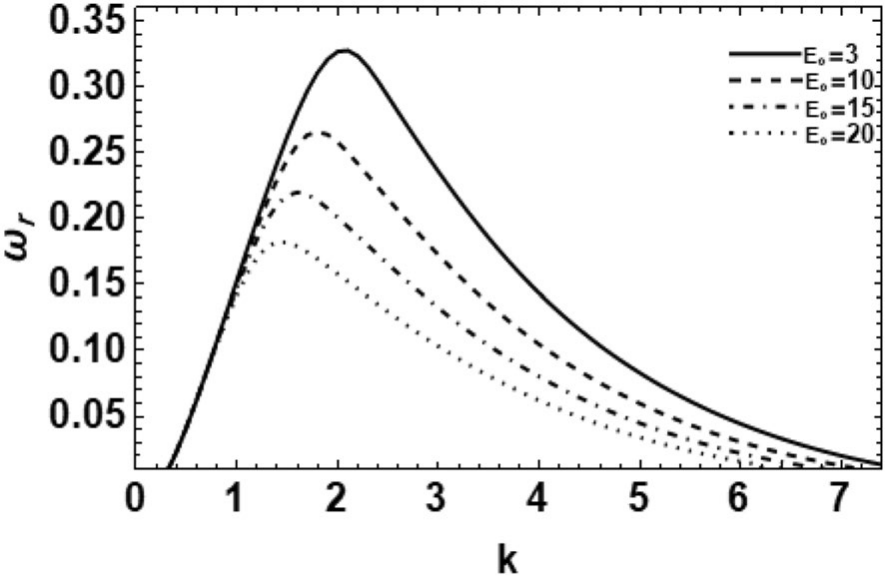
Shows the real part of the surface wave frequency against the wave numeral in light of Eq. (48) for different amounts of E_0_.

**Figure 9:**
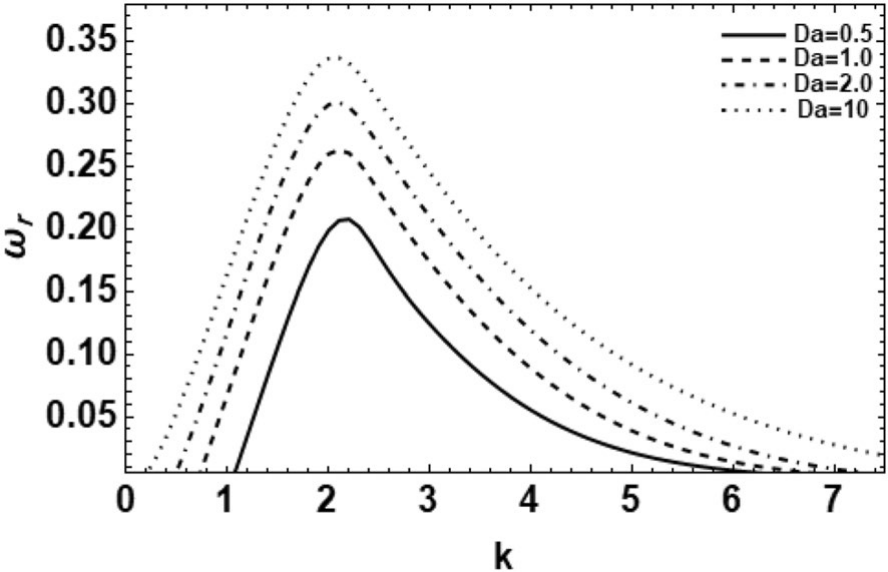
Shows the real part of the surface wave frequency against the wave numeral in light of Eq. (48) for different amounts of $Da$.

**Figure 10:**
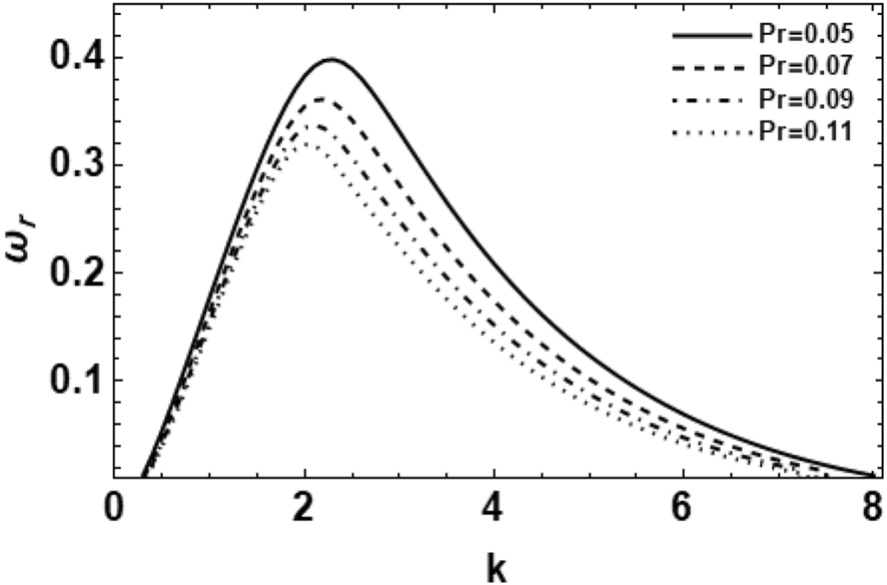
Depicts the real part of the surface wave frequency versus the wave number in light of Eq. (48) for different amounts of $\Pr$.

**Figure 11:**
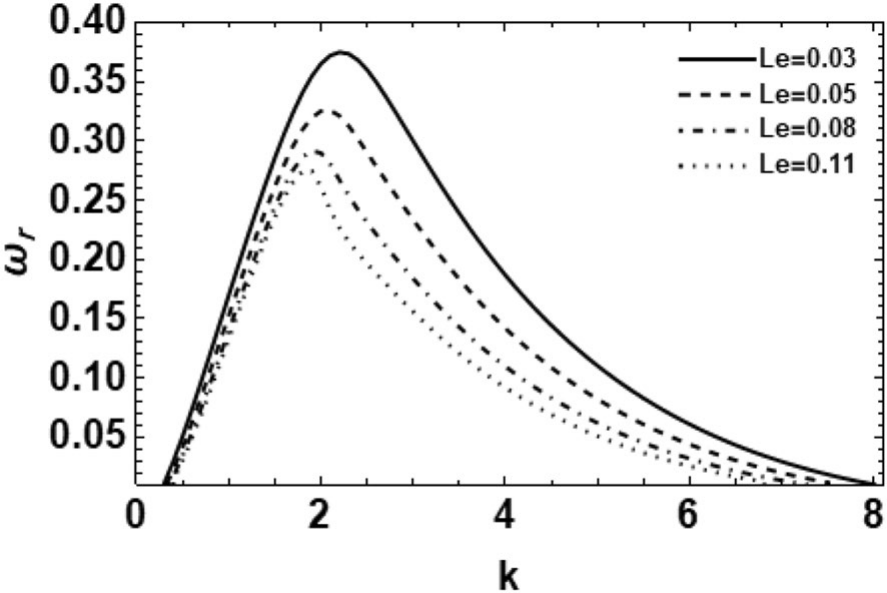
Shows the real part of the surface wave frequency against the wave number in light of Eq. (48) for different amounts of $Le$.

**Figure 12:**
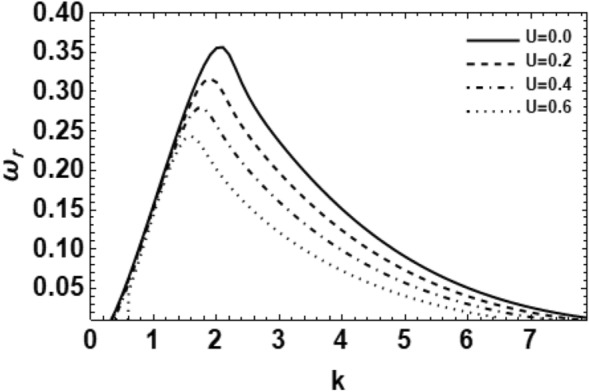
Shows the real part of the surface wave frequency against the wave number in light of Eq. (48) for different amounts of U.

**Figure 13:**
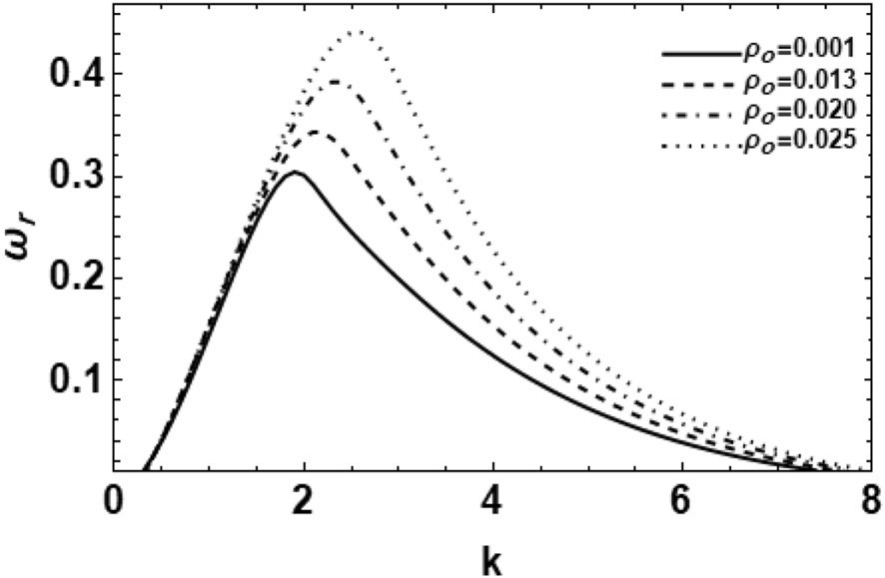
Depicts the real part of the surface wave frequency versus the wave number in light of Eq. (48) for various values of $\rho_{0}$.

**Figure 14:**
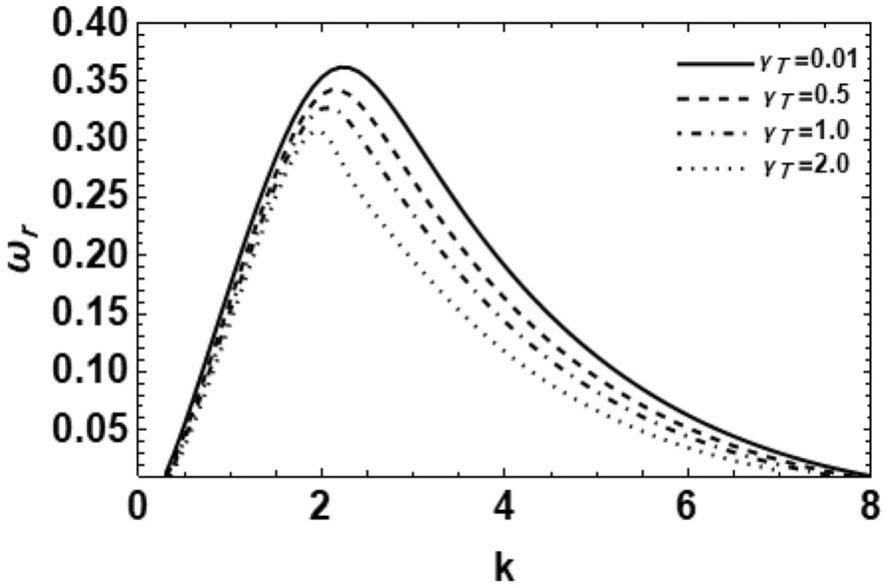
Depicts the real part of the surface wave frequency versus the wave number in light of Eq. (48) for different amounts of $\gamma_{T}$.

**Figure 15:**
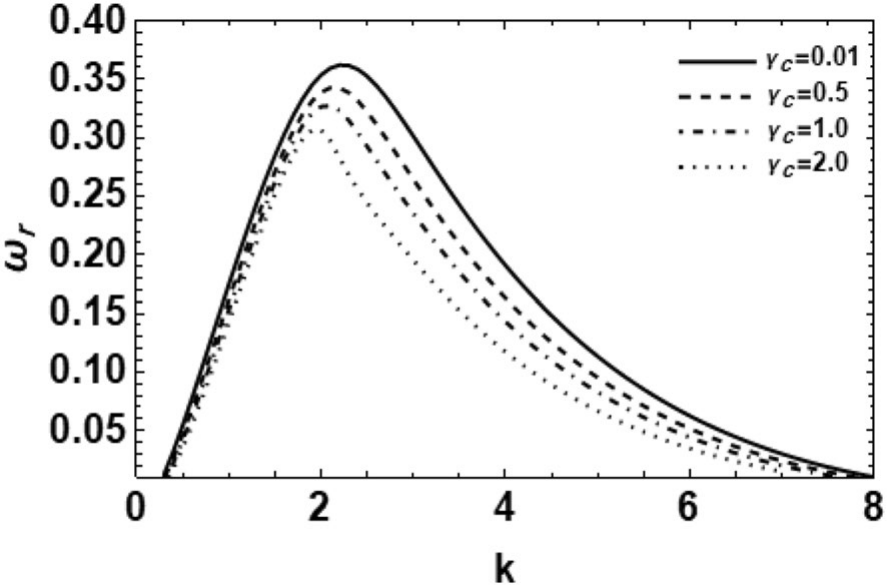
Depicts the real part of the surface wave frequency versus the wave number in light of Eq. (48) for different amounts of $\gamma_{C}$.

As exemplified in foregoing portion, the whole procedure of the non-dimensional dispersion connection is created. In the background of the time-based stability inspection, the frequency of waves has normally a complex performance, anywhere the real part establishes the perturbed growth rate, and the imaginary part signify the perturbed frequency. A closed analytical solution of the dispersion Eq. (48) is impracticable to be accomplished. Consequently, the MS might be employed to adopt the calculations. The study follows the early findings^26,30,32^. In light of the previous technique^33^, one might set $\omega_{i} = - k$, and usage $\omega_{r} = 0.03$ by approaching an initial estimation of the solutions. A replication of the solution of the regular couples $\left( {k,\omega_{r} } \right)$ is attained for various values of the diverse factors included in this analysis. Beforehand pictured a suitable graph, one could determine the applicable records. The subsequent methodology portrays a sequence of figures, ranging from Figs. 2, 3, 4, 5, 6, 7, 8, 9, 10, 11, 12, 13, 14 and 15. During these figures, the non-dimensional growing amounts are drawn versus the non-dimensional wave numeral of the surface waves. For additional suitability, the following factors are selected:

$$\,We = 1000,\,Z = 0.2,\,\,\,Da = 5,\,\Pr = 0.1,\,Le = 0.1,\,El = 0.5,\,Na = 0,\,Gr_{T} = 0.1,\,Gr_{C} = 0.3,\,E_{0} = 2.5,$$ $$\,\,\,\rho_{0} = 0.01,\,\eta = 0.5,\,\,\,\beta_{T} = 1,\,\beta_{C} = 1,\,c_{p} = 1,\,k_{f} = 1,\,D_{B} = 1,\,D_{T} = 1,\,\varepsilon = 0.5,\,U = 0,\,\lambda = 0.2\,$$ $$\,\,T_{a} = 1,\,C_{a} = 1,\,\gamma_{T} = 1\gamma_{C} = 1,\,a = 1,R = 0.1,\,{\text{and}}\,m = 1.$$

Figure 2 shows the non-dimensional growing rate $\omega_{r}$ versus the non-dimensional wave numeral $k$ of diverse amounts of the fluid Weber number for 3-dimensional configuration in case of $m = 1$. It is obvious that as $We$ increases, the growing amount disturbances stay identical at small standards of the wave number at approximately $k = 1.9$. Afterward, the instability influence appears and the extreme growth rate in addition to the dominant wave numbers also rises. Physically, due to the significance of the Weber numeral, it is clear that the rise of the Weber number can be generated by the rise of the fluid density and cylinder velocity, or by decreasing the ST. Significantly, as the fluid Weber numeral rises, the destabilizing range rises. Correspondingly, a larger liquid Weber number indicates that the effect of the ST is relatively small, i.e., the inertial force has an unstable effect on the gas-to-liquid interface. Consequently, the Weber numeral has a destabilizing influence. This outcome is well-matched with the findings of the preceding works^28^. It was showed that a growth in the fluid cylinder density or velocity have a destabilizing to the viscoelastic fluid cylinder. Consequently, one can decide that the viscoelastic fluid cylinder is destabilized definitely at large amounts of the fluid Weber numeral of specified conditions.

Figure 3 demonstrates the zero-shear viscosity impact on the growing rate disturbances through the Ohnesorge number. It is obvious that the growth amount as well as the instability zone increase with the increase of the Ohnesorge numeral $Z$. This confirms that the Ohnesorge numeral $Z$ has an instability influence on the stability map of the model. It is worthy to remember that the Ohnesorge number generally has a stabilizing outcome (stability numeral), that the viscosity prevents the liquid jet from the breakup. This result appeared in many works^28^, but the effect has been reflected here. Our results are compatible with some previous works^27^. Moatimid et al.^27^ has been discussed the stability map in the presence of MHT using the energy and concentration equations and found that the Ohnesorge numeral has destabilizing influence on the separated interface. This may be due to the presence of MP effect. Additionally, it was found a double role of the Ohnesorge number^19^. It is also noticed that the Ohnesorge number $Z$ stands for the proportion between the viscous and ST forces, and that a fewer Ohnesorge numeral generates a slighter viscous force in relationship by the ST force. In this situation, the growing amount is lesser. Consequently, the main and the upper cutoff wave numbers increase through the rise of the Ohnesorge number $Z$.

Figure 4 displays the influence of the elasticity number $El$ on the growth rate of wave of the asymmetric disturbances ($m = 1$) of a viscoelastic fluid cylinder against the wave numeral $k$. It is noticed that the wave growing amounts drop as the fluid elasticity number increases. Therefore, the elasticity numeral $El$ has a clear stabilizing effect. Not only does the elasticity numeral $El$ have a stabilizing influence, but also it has a weak destabilizing impact on the given system, and this consequence appears in the wave number range $3.5 \le k \le 5$. The destabilizing effect of the elasticity numeral $El$ was previously proved^28^. Lastly, one can conclude that the elasticity numeral $El$ demonstrates a dual role in the stability picture since it stabilizes and destabilizes the viscoelastic liquid jets. Once the elasticity numeral goes to zero i.e.,$El \to 0$, the growing amount disturbance of a viscoelastic liquid cylinder is changed to that of a Newtonian one, i.e., the flow has become Newtonian at this situation. Therefore, it is found that the liquid elasticity tends to increase the stability profile of the perturbation in viscoelastic fluid jets. In addition, the instability behavior of viscoelastic jets is influenced by the interaction of liquid viscosity and elasticity, in which the viscosity tends to dampen the instability, whereas the elasticity results in an enhancement of instability^34^.

The impact of the ratio of deformation retardation to stress relaxation time $\lambda$ on the wave growing amount is given in Fig. 5, where the time unchanging ratio $\lambda$ rises from 0.2 to 0.9. It is shown that the wave growing number of disturbances increases as the time constant ratio rises; nevertheless, the cutoff wave numeral does not change with the time unchanging ratio. Consequently, one concludes that the ratio of distortion retardation to stress relaxation time $\lambda$ has a destabilizing impact on the model. It must be noticed that El-Sayed et al.^28^ has confirmed the stabilizing effect of the time constant ratio $\lambda$ in his study. If one looks to Fig. 5, it is observed that an infinitesimal stabilizing effect occurs in the range $3 \le k \le 4$, which agrees with Ref.^28^. Relatively, the effect of the ratio of deformation retardation to stress relaxation time on the stability of viscoelastic jets is weak. Accordingly, one can find a double role of the time unchanging ratio $\lambda$ in the stability profile.

The influence of the thermal Grashof numeral $Gr_{T}$ on the wave growing amount of the non-Newtonian fluid cylinder is presented in Fig. 6. It is found that the wave growing of disturbances as well as the instability zone increase with the rise of the thermal Grashof numeral $Gr_{T}$. Hence, one concludes that the thermal Grashof number  $Gr_{T}$ exerts a destabilizing influence on the viscoelastic fluid cylinder in the considered system. According to the physical stand, the thermal Grashof numeral  $Gr_{T}$ is known as the ratio concerning Buoyancy to the viscous forces. Note that, free convection is caused by a change in density of a fluid due to a temperature change or gradient. Usually the density decreases due to an increase in temperature and causes the fluid to rise. This motion is caused by the buoyancy force. The major force that resists the motion is the viscous force. The Grashof number is a way to quantify the opposing forces^32^. This indicates that the Buoyancy force enhances the instability of the viscoelastic fluid jet, which, in turn, speeds up the breakup process. Furthermore, the main and the upper cutoff wave numerals rise by the increase of the thermal Grashof number. Moatimid et al.^19^ found that the Rayleigh number has a destabilizing effect. It is known that the thermal Grashof number is related to both the Prandtl numeral and the Rayleigh numeral by the relation $Ra = Gr_{T} \Pr$. This means that at a fixed value of the Prandtl numeral, together the Rayleigh and the Grashof numerals have the same influence.

There is an analogous form of the Grashof number used in cases of natural convection mass transfer problems. In the case of mass transfer, natural convection is caused by concentration gradients rather than temperature gradients. Figure 7 shows the impact of concentration on the wave growth rate of the non-Newtonian liquid cylinder throughout the solutal Grashof numeral $Gr_{C}$. It is evident from this figure that as the solute Grashof numeral $Gr_{C}$ increases, the area of instability wave number and the maximum growing amount in addition to the main wave numeral rise. In other words, it is obvious that the rise of the solute Grashof numeral $Gr_{C}$ occurs by an increase in the solutal expansion coefficient $\beta_{CL}$ or by a decrease in the liquid viscosity $\eta_{L}$, but in this situation, the additional factors are held fixed i.e. the influence of viscosity remains constant. Therefore, one deduces that the solute expansion coefficient $\beta_{CL}$(concentration phenomena) improves the instability of the liquid cylinder in the given system. This result has a significant role in the jet breakup procedure.

It is needed to calculate the effect of the non-dimensional EF E_0_. Figure 8 depicts that when the dimensionless EF E_0_ rises, the growing amount disturbances are still identical at small values of the wave number, approximately at $k = 1$. Afterward, the stability influence definitely appears and the maximum growing amount in addition to the instability region decrease. This means that the EF ${\mathbf{E}}_{0}$ produces a stabilizing influence on the interface. Furthermore, the decrease of the EF E_0_ reduces both the dominant and upper cutoff wave numbers, but the lower cutoff wave numeral remains fixed. Overall, one says that the EF resists the atomization process. Similar results have been recently found^19,27^.

Figure 9 represents the influence of Darcy numeral $Da$ on the growing amount of the wave rate disturbances in the stability picture. By the increase of $Da$, both the maximum growing rate and the equivalent upper cutoff wave number increase. Moreover, the lesser cutoff wave numeral decreases, but the dominant wave number remnants an unchanged. According to the physical stand point, the Darcy numeral is well-defined as the medium permeability in the non-dimensional form $Da = \kappa /\xi^{2}$, where κ is permeability. Therefore, the medium permeability in addition to the Darcy numeral $Da$ produces a destabilizing impact. This phenomenon can be described in such a way that the rise of the amounts of $Da$ sources a rise in permeability of the permeable media which, in turn, facilitates the flowing velocity of the fluid. Additionally, when the flowing velocity rises, the instability of the system rises. In a few words, when the permeability of the medium rises, the holes of the permeable medium are actual huge, and the resistance of the medium may be ignored so that the flowing velocity can increase and cause instability to the system. This outcome is in respectable agrees with the results early obtained^35^.

Figure 10 shows the departure of the non-dimensional growth rate against the dimensionless wave numeral for various amounts of the Prandtl numeral $\Pr$. As presented, as the amounts of the Prandtl number rise, the wave growing amount disturbances and the collection of instability wave number decrease intensely. Moreover, by growing the Prandtl number, the upper cutoff in addition to the dominant wave numbers decreases, while the lower cutoff wave number rises. From a physical interpretation, the Prandtl numeral is recognized as the ratio of kinematic viscosity (momentum diffusivity) to thermal diffusivity. Therefore, the rise of the Prandtl numeral may happen with an increase in the momentum diffusivity or a decrease in the thermal diffusivity. Consequently, one concludes that the momentum diffusivity has a stabilizing bearing on the considered scheme. This consequence is well-matched^19^. Note that whereas the Reynolds number and Grashof number are subscripted with a scale variable, the Prandtl number contains no such length scale and is dependent only on the fluid and the fluid state. The Prandtl number is often found in property tables alongside other properties such as viscosity and thermal conductivity.

Figure 11 shows the impact of thermal diffusivity on the wave growth rate of the non-Newtonian liquid cylinder through the Lewis number $Le$. It is seen that the effect of the Lewis number $Le$ on the rising number of waves on the viscoelastic jet is also significant in the same way as the effects of Prandtl numeral. Additionally, it is noticed that when the Lewis numeral $Le$ is slight, the wave growing amount of asymmetric disturbance on the viscoelastic jet becomes higher. This means a quick atomization and breakup. Therefore, one concludes that the Lewis number $Le$ stabilizes the considered system. Additionally, when the values of the Lewis numeral increase, the instability range and the highest growth rate disturbances reduce. By the same token, the upper cutoff and the main wave numbers reduce, while the lower cutoff wave number increases. Physically, Lewis numeral is recognized as the ratio of thermal diffusivity to mass diffusivity. It is used to characterize fluid flows where there is simultaneous HMT. The Lewis number puts the thickness of the thermal boundary layer in relation to the concentration boundary layer. Therefore, the improvement of the Lewis number may be generated by an increase in the thermal diffusivity or a decrease in the mass diffusivity. The rise in the Lewis numeral indicates a decrease in the Brownian movement of the liquid. Therefore, one concludes that the decrease in the Brownian motion develops the stability of the interface concerning the gas and the fluid. Moatimid et al.^19^ obtained similar conclusions.

It is necessary to explain the impact of the velocity ratio ${\mathbf{U}}$ on the wave growing amount for asymmetric disturbances ($m = 1$) as shown in Fig. 12. It is comprehensible that when the velocity ratio ${\mathbf{U}}$ rises, the growing amount disturbance stays applicable at minor values of the wave numeral, which is approximately at $k = 1.2$. After that, the stable influence appears definitely, and the maximum growing amount as well as the unstable range decrease. This means that the velocity ratio ${\mathbf{U}}$ has a stabilizing influence on the interface. Furthermore, the growing gas to liquid velocity ratio ${\mathbf{U}}$ reduces both the dominant and upper cutoff wave numerals, nonetheless the lower cutoff wave number remnants an unchanged. Overall, one can say that the velocity ratio $U$ resists the atomization process. Since the liquid Weber number is fixed here, then this influence is due to the ambient gas velocity i.e. the ambient gas velocity has stabilizing effect. Similar results have been recently found^14^.

The influence of the density ratio $\rho_{0}$ on the wave growing rate $\omega_{r}$ is inspected in Fig. 13. It is detected that when the density ratio rises, the growing amount disturbance stays applicable at smaller amounts of the wave numeral, which is approximately at $k = 1.1$. After this certain value, both the growth rate and the instability variety rise radically. Furthermore, the dominant wave numeral and the upper cut-off wave numbers also rise. Additionally, the lower cut-off wave numerals are constant due to the rise of the density ratio. Similarly, it can be found that Weber number $We$ has a fixed value in this graph, which involves that there is a rise in the gas density $\rho_{0G}$. Consequently, it might be decided that the rise of the gas density shows a vital role in the breakup procedure. In other words, the great ambient gas density develops the instability of the viscoelastic fluid cylinder. Consequently, the density ratio has a destabilizing bearing on the considered system, which corresponds to the early outcomes^36^.

In order to determine the impact of MHT through the MP influence on the development of the growing amount disturbances, one may plot the non-dimensional growth rate $\omega_{r}$ versus the non-dimensional wave number $k$ for some amounts of the heat and concentration parameters $\gamma_{T} \,\,\,{\text{and}}\,\,\,\gamma_{C}$. These computations are presented in Figs. 14 and 15, correspondingly. It is understood that the temperature or concentration coefficient describes the relative change of a physical property that is associated with a given change in temperature or concentration. It is clear that as the heat and concentration factors $\gamma_{T} \,\,{\text{and}}\,\,\gamma_{C}$ rise, the amounts of the growing number of perturbations and the upper cutoff in addition to the main wave numbers decay greatly. On the contrary, the amounts of the lower cutoff wave numbers rise with the rise in heat and concentration factors. In addition, these findings accord with those found previously^8,18^. They have established that the heat and concentration coefficients reduce the growing of the surface waves; consequently, they have a stabilizing impact. Generally, we may say that in the existence/nonexistence of energy/concentration equations, in view of the viscoelastic effect in the permeable medium, MHT has a stabilizing effect.

It should be noticed that the numerical calculations showed that the effect of the factors,$Na$,$T_{a}$ $D_{B}$ and $\eta$ on the stability diagrams is very weak and that their existence is approximately has no significant effect on the stability behavior. We are able to obtain a visualization of these effects, numerically through the Tables 1, 2, 3 and 4 at the same values of the other parameters as mentioned before. It is observed that by increasing the wave number $k$, the amounts of the growing amount of disturbances $\omega_{r}$ are rise at some amounts of the wave numeral $k$. After that, the performance is reflected in the decrease of the growing amount of disturbances $\omega_{r}$(vertical change in the tables). Furthermore, at a fixed amount of the wave numeral $k$ and by increasing the parameter values ($Na\,\,,\,\,D_{B} \,,\eta$), one finds that the growth rate of disturbances $\omega_{r}$ is also increases. Hence, the modified diffusivity ratio $Na$, the gas-to-liquid Brownian diffusion parameter ratio $D_{B}$ and the ratio of gas dynamic viscosity to zero shear viscosity of the liquid $\eta$ have a destabilizing impact on the stability outline. The previous results are compatible only with Tables 1, 3, and 4. In Table 2, and for some fixed values of the wave number $k$, it is obvious that the growing amount of disturbances $\omega_{r}$ reduces for small wave number amounts and then rises. Therefore, the temperature at the outer boundary $T_{a}$ has a double role in the stability configuration.

**Table 1:** The non-dimensional growing amount $\omega_{r}$ against the non-dimensional wave number $k$ for different amounts of the modified diffusivity ratio $Na$.

$k$	$\omega_{r}$ at $Na = 0$	$\omega_{r}$ at $Na = 5$	$\omega_{r}$ at $Na = 10$
0.1	− 0.049	− 0.018	− 0.367
1.1	0.176	0.261	0.307
2.1	0.327	0.507	0.590
3.1	0.224	0.510	0.644
4.1	0.135	0.345	0.464
5.1	0.078	0.212	0.292
0.1	0.041	0.123	0.173
7.1	0.018	0.066	0.096

**Table 2:** The non-dimensional growing amount $\omega_{r}$ against the non-dimensional wave number $k$ for different amounts of the temperature at the outer boundary $T_{a}$.

$k$	$\omega_{r}$ at $T_{a} = 1$	$\omega_{r}$ at $T_{a} = 5$	$\omega_{r}$ at $T_{a} = 10$
0.1	− 0.049	− 0.046	− 0.046
1.1	0.176	0.158	0.052
2.1	0.327	0.171	0.125
3.1	0.224	0.240	0.190
4.1	0.135	0.157	0.174
5.1	0.078	0.087	0.099
6.1	0.041	0.045	0.099
7.1	0.018	0.020	0.023

**Table 3:** The non-dimensional growth rate $\omega_{r}$ versus the non-dimensional wave number $k$ for various amounts of the gas-to-fluid Brownian diffusion parameter ratio $D_{B}$.

$k$	$\omega_{r}$ at $D_{B} = 1$	$\omega_{r}$ at $D_{B} = 3$	$\omega_{r}$ at $D_{B} = 10$
0.1	− 0.049	− 0.050	− 0.050
1.1	0.176	0.183	0.186
2.1	0.327	0.370	0.385
3.1	0.224	0.276	0.301
4.1	0.135	0.167	0.182
5.1	0.078	0.096	0.105
6.1	0.041	0.052	0.057
7.1	0.018	0.024	0.026

**Table 4:** The dimensionless growing amount $\omega_{r}$ against the dimensionless wave numeral $k$ for various amounts of the ratio of gas dynamic viscosity to zero shear viscosity of the liquid $\eta$.

$k$	$\omega_{r}$ at $\eta = 0.5$	$\omega_{r}$ at $\eta = 0.7$	$\omega_{r}$ at $\eta = 0.9$
0.1	− 0.0498	− 0.0494	− 0.0492
1.1	0.1761	0.1758	0.1754
2.1	0.3274	0.3282	0.3291
3.1	0.2246	0.2247	0.2248
4.1	0.1357	0.1359	0.1361
5.1	0.0781	0.0782	0.0783
6.1	0.0412	0.0413	0.0413
7.1	0.0182	0.0182	0.0182

## Concluding remarks

In our earlier study^18^, the EHD instability of a flowing dielectric viscous fluid cylinder with MHT was examined. Regardless of the short, ended construction of Hsieh’s formulation^5^, the current analysis has scrutinized the temperature in addition to the concentration fundamental equations. The motivation behind this study lies in the numerous applications of the non-Newtonian Oldroyd-B in geothermal and industrial developments as well as practical engineering. Consequently, the existing paper examines the linear EHD instability of a cylindrical interface that separates a moving viscoelastic fluid obeying Oldroyd-B and perfect liquid gas. For more clarification, abbreviations as well as nomenclature Section are provided at the beginning of the manuscript. Therefore, many symbols are ignored throughout all the processes. The methodology is considered in permeable media along with the effect of an unchanged axial EF. Undoubtedly, this meets real engineering applications. The interfacial tension is presumed to be as a profile of energy in addition to concentration. Therefore, the MP occurrence has been signified. The normal mode approach yields a complicated transcendental dispersion relation. Due to this complexity, the MS is utilized to attain a reasonable solution of the structure at hand. As widely understood, non-dimensional physical quantities can be used to explore the background of fluid flow. In addition, they minimize the amount of variables required to define the procedure. These numbers are typically having physical connotations that aid in the explanation of many scientific events. Therefore, the impact of the obtained non-dimensional physical numbers is evaluated and validated by the other existing works in the literature. When the results are compared with the present references, realistic deductions are reached. Generally, the following arguments characterize the main findings of the study:

Regardless of the previous works^7,28^, the current work indicates a dual influence on the stability profile in the reflected model.

- A fluid cylinder in a non-permeable medium has an additional unstable role rather than in a porous one.
- A liquid jet in the existence of MHT through MP effect makes the viscoelastic liquid jet more stable than in its absence.
- The EF $E_{0}$, Prandtl number $\Pr$, Lewis number $Le$, and gas-to-liquid velocity ratio $U$ have a stabilized influence.
- By using the BA on the streaming liquid jet, one obtains two non-dimensional numbers, thermal and solutal Grashof numbers $Gr_{T} \,\,\,{\text{and}}\,\,\,Gr_{C}$. These two numbers have a destabilizing effect.

### Supplementary Information

Supplementary Information.

## Data Availability

All data generated or analyzed during this study are included in this manuscript.

## Abbreviations

EHD: Electrohydrodynamics
EF: Electric field
MHT: Mass and heat transfer
RTI: Rayleigh–Taylor instability
MP: Maranogoni phenomenon
MHD: Magnetohydrodynamics
MS: Mathematica Software
KHI: Kelvin–Helmholtz instability
VPT: Viscous potential theory
BA: Boussinesq approximation
BCs: Boundary conditions
MF: Magnetic field
ST: Surface tension

## List of symbols

$$(r,\,\theta ,\,z)$$: Cylindrical coordinates
$a$: Outer cylindrical radius
$R$: Undisturbed cylindrical radius
$$T$$: Temperature
$$C$$: Concentration
$C_{a}$: Concentration at the outer boundary
$T_{a}$: Temperature at the outer boundary
$U_{L}$: Uniform inner stream
$U_{G}$: Uniform outer stream
$c.\,c.$: Preceding complex conjugate
$k$: Axial wave number
$m$: Azimuthal wave number
$$c_{p}$$: Specific heat
$$k_{f}$$: Thermal conductivity
$$g$$: Gravitational acceleration
$P_{j}$: Total perturbed pressure
$\tilde{V}_{j}$: Perturbed velocity for liquid and gas
$$We$$: Weber numeral
$$Z$$: Ohnesorge numeral
$$Da$$: Darcy numeral
$Gr_{T}$: Thermal Grashof numeral
$Na$: Modified diffusivity ratio

## Greek symbols

$\rho_{L}$: Inner liquid density
$\rho_{G}$: Outer gas density
$\gamma_{T}$: ST coefficient due to temperature
$\gamma_{C}$: ST coefficient due to concentration
$\varepsilon_{L}$: Inner liquid dielectric constant
$\varepsilon_{G}$: Outer gas dielectric constant
$$\sigma$$: ST coefficient
$$\sigma_{0}$$: Initial ST
$$\lambda$$: Wave length
$\omega$: Frequency of surface waves
$\omega_{r}$: Real part of the frequency
$\omega_{i}$: Imaginary part of the frequency
$$\xi$$: Interfacial displacement
$\xi_{0}$: Original significance of interfacial displacement
$\kappa$: Permeability of media
$\rho c_{p}$: Heat capacity of fluid
$\lambda_{1} ,\,\,\lambda_{2}$: Two characteristic parameters of the viscoelastic fluids
$\eta_{L}$: Zero shear viscosity
$Gr_{C}$: Solutal Grashof numeral
$$\Pr$$: Prandtl numeral
$$Le$$: Lewis numeral
$$El$$: Elasticity numeral
$$E_{0}^{2}$$: Bond EF

## Subscripts

$L$: Inner cylindrical flows
$G$: Upper cylindrical flows

## References

[1] El-Sayed MF, Moatimid GM, Elsabaa FMF, Amer MFE (2016). Ehectrohydrodynamic instability of a non-Newtonian dielectric liquid jet moving in a streaming dielectric gas with a surface tension gradient. Atom. Sprays.

[2] Fetecau C, Kannan K (2015). A note on an unsteady flow of an Oldroyd-B fluid. Int. J. Math. Math. Sci..

[3] Sajjadi H, Atashafrooz M, Amiri DA, Wang Y (2021). The effect of indoor heating system location on particle deposition and convection heat transfer: DMRT-LBM. Comput. Math. Appl..

[4] Moatimid GM, Zekry MH, Ibrahim DA (2023). Nonlinear EHD instability of two viscoelastic fluids under the influence of mass and heat transfer. Sci. Rep..

[5] Hsieh DY (1978). Interfacial stability with mass and heat transfer. Phys. Fluids.

[6] Nayak AR, Chakraborty BB (1984). Kelvin–Helmholtz stability with mass and heat transfer. Phys. Fluids.

[7] Fu QF, Deng XD, Jia BQ, Yang LJ (2018). Temporal instability of a confined liquid film with heat and mass transfer. AIAA J..

[8] Moatimid GM, Hassan MA, Mohamed MAA (2020). Temporal instability of a confined nano-liquid film with the Marangoni convection effect: Viscous potential theory. Microsyst. Technol..

[9] Moatimid GM, Mostafa DM (2023). Nonlinear stability of two dusty magnetic liquids surrounded via a cylindrical surface: Impact of mass and heat spread. Sci. Rep..

[10] Nayfeh AH (1972). Stability of liquid interfaces in porous media. Phys. Fluids.

[11] Li XG, Tankin RS (1991). On the temporal instability of a two-dimensional viscous liquid sheet. J. Fluid Mech..

[12] Fu QF, Yang LJ, Tong MX, Wang C (2014). Absolute and convective instability of a confined swirling annular liquid layer. Atom. Sprays.

[13] Le QH, Hussain Z, Khan N, Zuev S, Javid K, Khan SU, Abdelmalek Z, Tlili I (2023). Chebyshev collocation simulations for instability of Hartmann flow due to porous medium: A neutral stability and growth rate assessment. Ain Shams Eng. J..

[14] Moatimid GM, Mostapha DR (2023). EHD stability of a cylindrical boundary separating double Reiner–Rivlin fluids. Sci. Rep..

[15] Pascal H (1986). Stability of a moving interface in porous medium for non-Newtonian displacing fluids and its applications in oil displacement mechanism. Acta Mech..

[16] El-Dib YO, Moatimid GM (2004). Non-linear stability of an electrified plane interface in porous media. Z. Naturforsch..

[17] Cardenas MB (2015). Hyperorheic zone hydrologic science: A historical account of its emergence and a prospectus. Water Resour. Res..

[18] Amer MFE, Moatimid GM (2019). Electrohydrodynamic instability of a streaming dielectric viscous liquid jet with mass and heat transfer. Atom. Sprays.

[19] Moatimid GM, Amer MFE, Mohamed MAA (2021). Electrohydrodynamic instability of a cylindrical interface: Effect of the Buoyancy thermo-capillary in porous media. Micrograv. Sci. Technol..

[20] Chandrasekhar S (1961). Hydrodynamic and Hydromagnetic Stability.

[21] Fu Q-F, Yang L-J, Chen P-M, Liu Y-X, Wang C (2013). Spatial-temporal stability of an electrified viscoelastic liquid jet. J. Fluids Eng..

[22] Li F, Gañán-Calvo AM, López-Herrera JM (2013). Absolute and convective instability of a charged viscoelastic liquid jet. J. Nonnewton. Fluid Mech..

[23] Melcher JR (1963). Field Coupled Surface Waves.

[24] Bird RB, Armstrong RC, Hassager O (1977). Dynamics of Polymeric Liquids, 1: Fluid Mechanics.

[25] Hirata SC, Goyeau B, Gobin D (2009). Stability of thermosolutal natural convection in superposed fluid and porous layers. Transp. Porous Media.

[26] Bringedal C. Linear and nonlinear convection in porous media between coaxial cylinders, M. Sc. Thesis in Applied and Computational Mathematics, Department of Mathematics, University of Bergen (2011).

[27] Moatimid GM, Amer MFE, Mohamed MAA (2022). EHD azimuthal instability of two rigid-rotating columns with Marangoni effect in porous media. Indian J. Phys..

[28] El-Sayed MF, Moatimid GM, Elsabaa FMF, Amer MFE (2016). Axisymmetric and asymmetric instabilities of a non-Newtonian liquid jet in an inviscid streaming gas through media. J. Porous Media.

[29] Li X (1995). Mechanism of atomization of a liquid jet. Atomization Sprays.

[30] Furlani EP (2004). Temporal instability of viscous liquid microjets with spatially varying surface tension. J. Phys. A..

[31] Moatimid GM, Zekry MH (2020). Nonlinear stability of electro-viscoelastic Walters' B type in porous media. Microsyst. Technol..

[32] Bird RB, Stewart WE, Lightfoot EN (2002). Transport Phenomena.

[33] Gaster MA (1962). Note on the relation between temporally-increasing and spatially-increasing disturbances in hydrodynamic stability. J. Fluid Mech..

[34] Liu Z, Liu Z (2008). Instability of a viscoelastic liquid jet with axisymmetric and asymmetric disturbances. Int. J. Multiph. Flow.

[35] Moatimid GM, Amer MFE (2021). EHD instability of two rigid rotating dielectric columns in porous media. Pramana-J. Phys..

[36] Brenn G, Liu Z, Durst F (2000). Linear analysis of the temporal instability of axisymmetrical non-Newtonian liquid jets. Int. J. Multiph. Flow..

