# Supplementary material for: The influence of energy and temperature distributions on EHD destabilization of an Oldroyd-B liquid jet
Source: Sci Rep. 2023 Sep 26;13:16118. doi: 10.1038/s41598-023-43157-z (PMC10522636; doi:10.1038/s41598-023-43157-z)
Supplement: Supplementary file 1 — Supplementary Information. [file 41598_2023_43157_MOESM1_ESM.docx]

**APPENDIX**

- The values of the constants that are given in Eqs. (10), and (21) may be listed as follows:

$A_{j}=\frac{\Delta_{jT}}{\Delta_{T}}T_{0}, j=1, 2, 3$, where

,

, and

- The values of the constants that are given in Eqs. (12), and (22) may be registered as follows:

$B_{j}=\frac{\Delta_{jC}}{\Delta_{C}}C_{0}, j=1, 2, 3$, where

, ,

,

and .

- The following is a list of the values of the constants $D_{1} , D_{2}$ and $D_{3}$ that appears in Eqs. (18), (19) and (20):

$D_{1}=\frac{1}{\Delta}\left(ik\sigma_{0}H(R)\Delta_{11}+\frac{\rho_{0L}g}{k}\Delta_{12}\right)$, $D_{2}=\frac{1}{k\eta\left(\omega\right)\Delta}\left(ik\sigma_{0}H(R)\Delta_{21}+\frac{\rho_{0L}g}{k}\Delta_{22}\right)$,

and $D_{3}=\frac{1}{k\eta\left(\omega\right)\Delta}\left(ik\sigma_{0}H(R)\Delta_{31}+\frac{\rho_{0L}g}{k}\Delta_{32}\right)$,

where

$\Delta=\left|\begin{matrix} \Gamma_{11} & \Gamma_{12} & \Gamma_{13} \\ \Gamma_{21} & \Gamma_{22} & \Gamma_{23} \\ \Gamma_{31} & \Gamma_{32} & \Gamma_{33} \end{matrix}\right|$, $\Delta_{11}=\left|\begin{matrix} 1 & \Gamma_{12} & \Gamma_{13} \\ m/kR & \Gamma_{22} & \Gamma_{23} \\ 0 & \Gamma_{32} & \Gamma_{33} \end{matrix}\right|$, $\Delta_{12}=\left|\begin{matrix} \Gamma_{1} & \Gamma_{12} & \Gamma_{13} \\ \Gamma_{2} & \Gamma_{22} & \Gamma_{23} \\ \Gamma_{3} & \Gamma_{32} & \Gamma_{33} \end{matrix}\right|$, $\Delta_{21}=\left|\begin{matrix} \Gamma_{11} & 1 & \Gamma_{13} \\ \Gamma_{21} & m/kR & \Gamma_{23} \\ \Gamma_{31} & 0 & \Gamma_{33} \end{matrix}\right|$,

$\Delta_{22}=\left|\begin{matrix} \Gamma_{11} & \Gamma_{1} & \Gamma_{13} \\ \Gamma_{21} & \Gamma_{2} & \Gamma_{23} \\ \Gamma_{31} & \Gamma_{3} & \Gamma_{33} \end{matrix}\right|$, $\Delta_{31}=\left|\begin{matrix} \Gamma_{11} & \Gamma_{12} & 1 \\ \Gamma_{21} & \Gamma_{22} & m/kR \\ \Gamma_{31} & \Gamma_{32} & 0 \end{matrix}\right|$, and $\Delta_{23}=\left|\begin{matrix} \Gamma_{11} & \Gamma_{12} & \Gamma_{1} \\ \Gamma_{21} & \Gamma_{22} & \Gamma_{2} \\ \Gamma_{31} & \Gamma_{32} & \Gamma_{3} \end{matrix}\right|$.

Where

$\Gamma_{11}=\frac{2ik^{2}}{\left(k^{2}-q_{L}^{2}\right)}I'_{m}(kR)$, , $\Gamma_{13}=\frac{im}{q_{L}R}$,

$H(R)=\gamma_{T}T_{L}(R)+\gamma_{C}C_{L}(R)$,., $\Gamma_{22}=\frac{m-1}{q_{L}R}I_{m-1}(q_{L}R)+I'_{m}(q_{L}R)$,

, .

$\Gamma_{31}=\frac{-2mk}{R\left(k^{2}-q_{L}^{2}\right)}I_{m}(kR)$, $\Gamma_{32}=i\left(\frac{k}{q_{L}}I_{m-1}(q_{L}R)+\frac{m}{kR}I_{m}(q_{L}R)\right)$, $\Gamma_{33}=-I'_{m}(q_{L}R)$,

$\Gamma_{3}=\frac{-m}{kR}\left(\alpha_{2}I_{m}(S_{L}R)+\alpha_{3}I_{m}(n_{L}R)\right)+\frac{im}{k^{3}R}\left(S_{L}^{2}I_{m}(S_{L}R)\alpha_{2}+n_{L}^{2}I_{m}(S_{L}R)\alpha_{3}\right)$.

- The values of the constants $D_{4}$and $D_{5}$that are appearing in Eqs. (23), (24) and (25) may be listed as follows:

,

and ,

where , , and

,

.

- The following is a list of the values of the constants $F_{1} , F_{2}$ and $F_{3}$ that appears in Eqs. (33) and (34):

$F_{1}=i(\varepsilon-1)E_{0}\xi_{0}\frac{\Delta_{15}}{\Delta_{E}} , F_{2}=i(\varepsilon-1)E_{0}\xi_{0}\frac{\Delta_{25}}{\Delta_{E}} , \text{and} F_{3}=i(\varepsilon-1)E_{0}\xi_{0}\frac{\Delta_{35}}{\Delta_{E}}$,

where

,,

, and.
